# Injectable ROS homeostasis protective hydrogel inhibiting microglial ferroptosis through the Nrf2/Slc7a11/Gpx4 to alleviate neuropathic pain and promote spinal cord injury repair

**DOI:** 10.1016/j.redox.2025.103816

**Published:** 2025-08-08

**Authors:** Lu Li, Yu Cao, Xiangsheng Zhang, Jiayi Guo, Ziqiang Lin, Pengyu Zhou, Chuyin Chen, Jiahao Chen, Yike Liu, Danzhi Luo, Jiurong Chen, Yingdong Deng, Peng Sun, Zhiwen Zeng, Jun Zhou

**Affiliations:** a Department of Anesthesiology, The Third Affiliated Hospital, Southern Medical University, Guangzhou, 510000, China; b Institute of Biological and Medical Engineering, Guangdong Academy of Sciences, Guangzhou, 510316, China; c Department of Ultrasound, Foshan Fosun Chancheng Hospital, Foshan, 528031, China; d Guangdong Key Lab of Medical Electronic Instruments and Polymer Material Products, Guangzhou, 510500, China; e Department of Anesthesiology, Sun Yat-sen University Cancer Center, Guangzhou, 510060, China; f State Key Laboratory of Oncology in South China, Collaborative Innovation Center for Cancer Medicine, Guangzhou, 510060, China; g ShunDe Hospital GuangZhou University of Chinese Medicine, Guangzhou, 510000, China

**Keywords:** Hydrogel, Quercetin, Microglial ferroptosis, Spinal cord injury, Neuropathic pain

## Abstract

Spinal cord injury (SCI) induced neuropathic pain (NP) remains a major clinical challenge due to persistent neuroinflammation and oxidative stress. We developed an injectable methacrylated and thiolated gelatin hydrogel loaded with quercetin (MSQ) to synergistically scavenge reactive oxygen species (ROS) and inhibit microglial ferroptosis for NP alleviation and neural repair. The MSQ hydrogel exhibited rapid photocrosslinking, sustained quercetin release, and robust ROS scavenging via thiol groups and quercetin, maintaining intracellular redox homeostasis. MSQ attenuated LPS-induced ferroptosis in BV2 microglia by upregulating Nrf2 expression, promoting its nuclear translocation, and activating the Slc7a11/Gpx4 pathway, thereby reducing lipid peroxidation and inflammatory cytokine release. Network pharmacology and molecular dynamics simulations confirmed quercetin's high-affinity binding to Nrf2. In a murine SCI model, MSQ implantation significantly reduced lesion area, suppressed microglial ferroptosis, and decreased pro-inflammatory mediators (TNFα, IL-1β, IL-6), while enhancing neuronal survival (Nissl/NeuN^+^ cells) and axonal regeneration (MAP2/5-HT^+^ expression). Motor functional recovery assays revealed improved BMS scores, gait regularity, and mechanical/thermal pain thresholds in MSQ-treated mice. This study highlights MSQ hydrogel as a multifunctional therapeutic platform that targets ROS homeostasis and microglial ferroptosis via the Nrf2/Slc7a11/Gpx4 axis, offering a promising strategy for post-SCI NP management and neural regeneration.

## Graphical abstract

**Figure:**
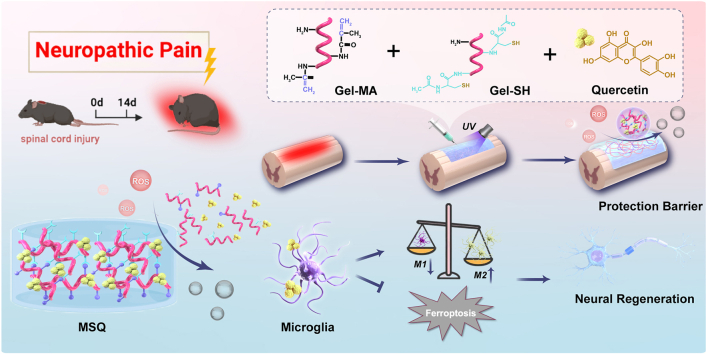

## 1 Introduction

Spinal cord injury (SCI) is a major debilitating condition that is frequently associated with spinal fractures and dislocations [1]. SCI results in the loss of sensory and voluntary motor function below the affected segment, causing substantial physical and psychological distress in patients worldwide [2]. The treatment of SCI is a considerable challenge and often complicated by high susceptibility to neuropathic pain (NP) [3]. Studies point out that up to 94 % of patients with SCI experience long-time pain, and with approximately 70 % developing NP [4]. Although the exact development mechanisms of post-SCI NP are not well understood, the neuroinflammation microenvironment including increased levels of inflammatory mediators, oxidative stress, and cell death, is the key to the development of NP [5]. Research proved that improvement of SCI microenvironment with bioactive drugs or factors can reduce secondary damage, alleviate NP, and facilitate neurological recovery [6]. Nevertheless, effective neural regeneration strategies for NP alleviation and motor function rehabilitation are still lacking. New treatments are crucial to address this widespread issue. Lately, advanced drug-delivery systems containing bioactive molecules from Chinese herbs with various pharmacological effects is a potential strategy for the treatment of NP and SCI [7].

Quercetin, a polyhydroxy flavonoid, shows promise for the treatment of post-SCI NP owing to its antioxidant, anti-inflammatory, and anti-apoptotic effects [8]. Literatures have reported that quercetin can inhibit the p38MAPK/iNOS signaling pathway [9], decrease malondialdehyde (MDA) levels [10], and boost superoxide dismutase activity, which reduces oxidative damage in rat models post-SCI [11]. Ali et al. demonstrated that quercetin inhibits T-type Cav channels and affects deubiquitylation, to reduce inflammation and migrate NP [10,12]. Nonetheless, the bioavailability and duration of quercetin's pharmacological effects at the site of SCI, as well as its mechanisms in alleviating NP and facilitating neural function recovery post-SCI, remain to be fully elucidated.

Microglia are the primary immune cells in the central nervous system (CNS) and crucial for the development of NP post-SCI [13]. Ferroptosis is a form of cell death driven by iron and lipid peroxide build-up. Microglial ferroptosis usually occurs when the mitochondrial dysfunction in microglia impairs cell viability. Microglial ferroptosis causes cell membrane rupture and releases inflammatory factors that induce iron build-up in other microglia to trigger ferroptosis [14,15]. This cycle worsens neuroinflammation and affects neuronal function and survival, thereby exacerbates NP post-SCI and hinders neural function recovery [16]. Strategies include reducing reactive oxygen species (ROS) to inhibit ferroptosis, protecting mitochondrial function, minimizing inflammatory factor release was proposed to alleviate NP and spinal cord repair. Notably, it is suggested that quercetin can modulate microglial phenotypes by facilitating the transition from the pro-inflammatory M1 state to the anti-inflammatory M2 state, thereby attenuating microglia-mediated inflammatory responses and inhibiting ferroptosis in spinal neurons [17]. Despite these findings, the precise occurrence of ferroptosis in microglia post-SCI and their subsequent roles remain inadequately elucidated [[18], [19], [20]].

Direct administration at SCI sites is compromised by the cerebrospinal fluid circulation, which dilutes the drug plasma concentration and prevents sustained therapeutic effects [21,22]. Given the clinical use of quercetin is restricted by its poor water solubility, short circulation half-life, low delivery efficiency and the risks of adverse reactions [[23], [24], [25]], various drug-delivery systems were proposed to delivery drug effectively. Hydrogels are widely utilized as drug-delivery vehicles, which endowed with three-dimensional crosslinked networks, high water content and the mimicking natural extracellular matrix (ECM) [26]. They often display excellent biocompatibility, and adjustable properties, making them ideal for biomedical applications such as drug-delivery, tissue engineering, and wound healing [27]. Hydrogels are made from natural biopolymers (e.g., collagen, gelatin, and chitosan) and synthetic polymers (e.g., polyethylene glycol, polyvinyl alcohol, and acrylonitrile) [28]. Gelatin, which is derived from collagen in biological tissues, is biodegradable, biocompatible, and easy modifying so that it widely applicable in biomedical materials [[29], [30], [31]]. Numerous reports indicated that hydrogels can effectively connect severed spinal cord tissues, fostering axonal growth and neuronic regeneration [32,33]. Furthermore, advanced hydrogels drug-delivery systems were fabricated to enable targeted and sustained release of pharmaceuticals, which enhances drug binding to target proteins, and reducing inflammation to accelerate tissue repair [[34], [35], [36]]. Considering the importance of neuron protection under inflammation, a therapeutic strategy combining quercetin targeted drug with protective hydrogel synergistic was proposed for the management of post-SCI NP and neural regeneration.

**Scheme 1:**
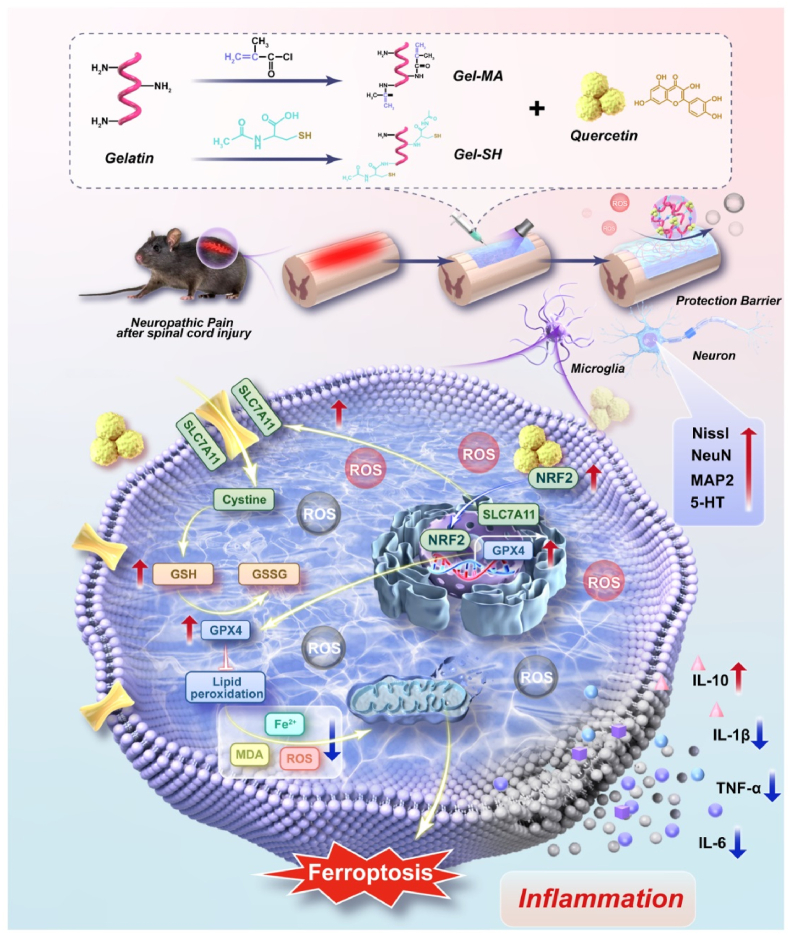
Schemetic of the preparation of injectable protective MSQ hydrogel with antioxidation, antiinflammtion and synergistic protective effect on local cells. MSQ hydrogel showed microglia ferroptosis inhibition to alleviate neuropathic pain and promote spinal cord injury repair through Nrf2/Slc7a11/Gpx4 pathway.

Therefore, in this study, as shown in Scheme 1, we developed an injectable quercetin-loaded hydrogel (MSQ) system based on functional gelatin. The functional gelatin was modified with methacrylate groups and thiol groups, respectively, and they were crosslinked to form hydrogels rapidly (<10 s) under ultraviolet irradiation. The residual reductive thiol groups of MSQ hydrogel can scavenge excess ROS synthetic with the sustained release of quercetin, this synergy protection effect of MSQ inhibited the pro-inflammation microenvironment, protecting the local cells and preserving its targeted drug-delivery efficacy. The enhanced antioxidant behaviors of MSQ were confirmed by the intracellular and extracellular ROS scavenging experiments. The degradation behaviors of MSQ hydrogel were measured in vitro and in vivo, and it proved the sustained release of quercetin mostly depends on the degradation. Cell counting kit-8 (CCK-8), lactate dehydrogenase (LDH), and propidium iodide (PI) staining tests identified that the MSQ hydrogel had good biocompatibility. Immunofluorescence staining, western blotting, quantitative polymerase chain reaction (qPCR), and other analyses revealed that the MSQ hydrogel significantly alleviated microglial ferroptosis and migrated inflammation. Furthermore, a mouse SCI model was constructed to evaluate MSQ hydrogels by histology, immunohistology, and molecular biology methods, and it revealed that the MSQ hydrogel reduced ROS, inflammation, ferroptosis, and promoted axonal regeneration at injury sites, resulting in alleviating post-SCI NP and facilitated motor function rehabilitation. This study further elucidated the molecular mechanism by network pharmacology and molecular dynamics analyses, it identified quercetin mitigates post-SCI NP by upregulating microglia Nrf2 expression to activates the Slc7a11/Gpx4 pathway so that it inhibits ferroptosis, and reduces the release of inflammatory mediators. These findings indicate that the MSQ hydrogel provides a potential strategy for post-SCI NP alleviation and accelerating spinal cord regeneration.

## 2 Materials and methods

### 2.1 Materials

Gelatin and *N*-acetylcysteine (NAC) were purchased from Merck Ltd. Quercetin and ML385 were obtained from MedChemExpress. Methacrylic anhydride, lithium phenyl-2,4,6-trimethylbenzoylphosphinate (LAP), 1-(3-dimethylaminopropyl)-3-ethylcarbodiimide hydrochloride (EDC·HCl), N-N-hydroxy succinimide (NHS), and the DPPH and ROS assay kits were obtained from Shanghai Aladdin Biochemical Technology Co., Ltd. Fetal bovine serum (FBS), Dulbecco's modified Eagle medium (DMEM), and a live/dead cell staining kit were purchased from Thermo Fisher Scientific. The CCK-8 and LDH assay kits were purchased from Dojindo Laboratories. MDA, reduced glutathione (GSH), and ferrous ions (Fe^2+^) were procured from the Nanjing Jincheng Bioengineering Institute. All commercially available reagents and solvents were used directly without further purification.

### 2.2 Synthesis of thiolated gelatin

Thiolated gelatin (Gel-SH) was prepared by the typical EDC reaction between NAC containing thiol groups and gelatin. Briefly, gelatin powder was added to deionized water in a flask while stirring at 60 °C to obtain a clear 5 wt% gelatin solution. NAC (25 mmol) was then added to the solution, and the pH was adjusted to 5. After cooling to room temperature, EDC·HCl (25 mmol) and NHS (25 mmol) were added to the reaction solution, and the reaction was allowed to proceed for 24 h. During the reaction, the pH of the reaction mixture was maintained at 5. After 24 h, the mixture was diluted to end the reaction and transferred to a dialysis tube (12–14 KDa cutoff). Gel-SH was obtained after dialysis and lyophilization. The Ellman's test was performed to determine the degree of thiol group substitution.

### 2.3 Synthesis of gelatin methacryloyl

Gelatin methacryloyl (Gel-MA) was synthesized as described previously. 10 wt% gelatin was mixed in phosphate-buffered saline (PBS) at 50 °C for 30 min till it was completely dissolved. Next, methacrylic anhydride was slowly added to the mixture, which was left to react for 3 h at 50 °C. Subsequently, the reaction solution was diluted with 50 mL of warm PBS and dialyzed against deionized water for 3 days. The deionized water was refreshed three times daily. Finally, Gel-MA was harvested by collecting the lyophilized product.

### 2.4 Preparation of the MSQ hydrogel

A precursor solution was prepared by dissolving10 wt% Gel-SH, 10 wt% Gel-MA, and 1 w/v% quercetin into 0.1 wt% LAP photo-initiator solution. The prepared solution was crosslinked under UV irradiation (15 W/m^2^, 365 nm, China) to form MSQ hydrogel. In addition, a MS hydrogel without quercetin was fabricated using the same method.

### 2.5 Material characterization

The synthesized Gel-SH and Gel-MA were analyzed using a Fourier transform infrared (FTIR) spectrometer (Thermo Fisher Scientific, USA) equipped with attenuated total reflection (ATR) and a Bruker Fourier transform (FT)-nuclear magnetic resonance (NMR) spectrometer (Bruker Analytik GmbH, Germany) at a frequency of 400 M Hz. H^1^ NMR spectra of Gel-SH and Gel-MA that dissoluted in deuterium oxide (D_2_O). Scanning electron microscopy (SEM) was performed to observe the morphologies of the prepared hydrogels. After the freeze-dried hydrogels were sputter-coated with 10 nm of gold/palladium, they were transferred to the sample stage and observed using a Phenom Prox Desktop SEM (Phenom, World Company, Netherlands).

### 2.6 Swelling and degradation behaviors

The swelling and degradation behaviors of the prepared hydrogels were evaluated using the weighting method. For evaluating the swelling behavior, freeze-dried hydrogel samples (n = 3) were weighed (*Wd*) and immersed in a PBS solution for 24 h to reach equilibrium. The weight of the swollen hydrogel was recorded (*Ws*) after the residual water on the surface was carefully removed. The equilibrium swelling ratio (ESR) is calculated using the following formula:

(1) ESR = (*Ws-Wd*)/*Wd* × 100 %.

For determining the degradation rate of the prepared hydrogel, the weight of the hydrogel samples (n = 3) was recorded at predetermined intervals after immersion in PBS solution at 37 °C. The s equilibrium welling ratio was set as the initial point (*Wi*), and the weight of the samples at predetermined times was recorded as *Wt*. The degradation rate was calculated using the following formula:

(2) Degradation rate = (*Wi-Wt*)*/Wi* × 100 %

### 2.7 Rheological properties of the MSQ hydrogel

The rheological measurements were performed using an Anton Paar rheometer (MCR 302, Anton Paar Co., Austria). The flow curve of the precursor solution of the hydrogel was obtained to study the viscosity over a range of shear rates from 0.1 to 100 s^−1^. The strain amplitude sweep test with strain amplitudes set from 0.01 % to 100 % was conducted to measure the linear viscoelastic region of the prepared hydrogels at 10 rad/s, and the frequency sweep test was also conducted with frequencies set from 0.1 to 100 rad/s to measure the rheological properties of the prepared hydrogels. After the prepared sample was placed into the gap of 25-mm parallel plates, the loss modulus (G″) and storage modulus (G′) were recorded. All the experimental tests were performed at 37 °C, and the gap distance was set to 1 mm.

### 2.8 Release behaviors of the MSQ hydrogel

The MSQ hydrogel samples (n = 3) were weighed (W0) and immersed in PBS at pH 7.4, and then they were maintained under agitation at 37 °C. On the first day, aliquots of the release medium were collected at intervals of 0, 2, 4, 6, 8, and 24 h, followed by additional sampling on days 2, 4, 6, and 8. The absorbance at 374 nm wavelength, which corresponds to the quercetin absorption peak, was determined using a UV spectrophotometer to detect the content of quercetin releasing from MSQ hydrogel. Subsequently, the drug-release profile was constructed for the assessment of the release characteristics and kinetics of quercetin from the hydrogel. The cumulative release rate of quercetin was calculated using (Wt/W0∗1 %) ∗100 %. Wt was the cumulative release content of quercetin at t timepoint; W0∗1 % was the totle quercetin content incorporated into the MSQ hydrogels.

### 2.9 Antioxidation evaluation

DPPH free radical-scavenging assay, Superoxide anion (·O_2_^−^) scavenging ability investigation and intracellular total ROS activity assay kit were performed to study the free radical resistance of the prepared hydrogels. For DPPH free radical-scavenging assay, 100 μM and 200 μM DPPH ethanol solutions were incubated with the testing samples in the dark for 30 min. After 30 min of incubation, the absorbance of the sample solutions was measured at a wavelength of 517 nm to assess the DPPH-scavenging behavior of the prepared Gel-MA, MS, and MSQ hydrogels. The DPPH-scavenging ratio (%) was calculated as follows:

(3) DPPH-scavenging ratio (%) = (A_blank_ - A_control_)/A_blank_ × 100 %

Where A_blank_ and A_control_ were the absorbance values of the blank and sample groups (hydrogel) at 517 nm, respectively.

For superoxide anion (·O_2_^−^) scavenging ability investigation, pyrogallol can undergo autoxidation to produce ·O_2_^−^ and colored intermediates whose absorption intensity is proportional to the amount of ·O_2_^−^ under weakly basic conditions. Therefore, pyrogallol was used to verify the ·O_2_^−^ scavenging activity of the prepared hydrogels. The Tris-HCl buffer (pH 8.5) were incubated with the testing samples, then pyrogallol (final concentration 250 μM) was added and incubated for 20 min.The absorbance of pure pyrogallol solution (Ap), a mixed solution of pyrogallol and the prepared hydrogels (As) were measured at 319 nm. The ·O_2_^−^ inhibition rate (%) was calculated as follows:

(4) O_2_^−^ inhibition rate (%) = [(Ap - As)/As] × 100 %

Furthermore, an active oxygen species assay kit was used to evaluate the intracellular ROS-scavenging behavior of the prepared hydrogels. After BV2 cells (5 × 10^4^ cells/well) were cultured for 24 h, the culture medium was replaced with 100 μM DCFH-DA serum-free medium. The test samples were then cultured for another 30 min. After 30 min, the BV2 cells were treated with a medium containing 10 % FBS, Rosup agent, and the prepared hydrogel extracts for 30 min. Finally, the stained cells were observed and imaged using a fluorescence microscope (BX53; Olympus, Japan).

### 2.10 Cell cultures and treatments

The immortalized BV2 murine microglia cells (CL-0493, Prooell Life Science & Technology Co., China) were cultured in complete culture medium that containing Dulbecco's modified Eagle's medium (DMEM; Invitrogen, Camarillo, CA), supplemented with 10 % (v/v) heat-inactivated fetal bovine serum (FBS, ExCell, FSP500), streptomycin (100 μg/mL), and penicillin (100 U/mL) at 37 °C under a humidified atmosphere with 5 % CO2. BV2 cells were seeded at a density of 2 × 10^5^ cells/mL and treated with 10 μM quercetin or 8 μM ML385 for 2 h, with the dosage regimen based on the previous studies [37,38]. The cells were then stimulated with Lipopolysaccharide (LPS, Sigma-Aldrich, L2880, 1 μg/ml) for 8 h.

### 2.11 Plasmid transfection

The mammalian expression plasmids containing Nrf2-WT, or Nrf2-MUT were purchased from Tsingke Biotechnology (Beijing, China), with the sequence of Nrf2-WT and Nrf2-MUT were shown in Supplemental Table S1. The BV2 cells were transfected using Lipo3000 reagent (Invitrogen, USA), according to the manufacturer's instructions. Briefly, 2 μl Lipo3000 reagent and 2 μl plasmid (0.8 μg) were diluted into 50 μl Opti-MEM and incubated at room temperature for 5 min before mixing and incubation for a further 20 min.

### 2.12 Cell counting Kit-8 assay (CCK-8)

CCK-8 assay was used to study the biocompatibilities by extracts method. Hydrogel samples were sterilized and incubated with 10 ml of complete culture medium at 37 °C for 24 h. The resulting extracts were filtered through a 0.22 μm filter and stored at −20 °C. BV2 cells (1 × 10^4^) were seeded in 96-well plates and cultured with hydrogel extracts for 1, 4, and 7 days. The cells cultured with complete culture medium was regarded as control group. On the assay day, BV2 cells were washed with PBS, treated with CCK-8 solution, and then incubated for 1 h. Absorbance was measured at 450 nm using a multi-mode microplate reader (Synergy HTX, Bio-Tek, USA) to assess cell viability, and the data obtained were subsequently analyzed for statistical significance using Prism software.

### 2.13 Lactate dehydrogenase assay (LDH)

To assess cell membrane integrity and cytotoxicity, an LDH assay was performed using the Cytotoxicity LDH Assay Kit (CK12, Dojindo Molecular Technologies, Japan). BV2 cells were seeded at a density of 1 × 10^4^ cells per well in a 96-well plate and cultured for 1, 4, or 7 days with complete culture medium, MS hydrogel extract, or MSQ hydrogel extract.100 μL of working solution was added directly to each well. The plate was incubated at room temperature for 30 min to allow the reaction to proceed. The reaction was stopped by adding 50 μL of stop solution to each well. Absorbance was measured at 490 nm using a multi-mode microplate reader, and the data obtained were subsequently analyzed for statistical significance using Prism software.

### 2.14 Hemolysis test

Fresh anticoagulated rat blood was added to 10 mL of normal saline and centrifuged at 1500 rpm for 15 min to collect red blood cells (RBCs). Next, the collected RBCs were diluted with normal saline to adjust the absorbance of the RBC solution to 0.8 ± 0.3 at a wavelength of 545 nm. After pretreating the samples (n = 3) for 1 h with normal saline, the prepared RBC diluent solution was added, and the samples were incubated at 37 °C for 30 min. Normal saline was used as the negative control, whereas 0.1 % Triton solution was used as the positive control. The incubated RBC diluent solution was centrifuged at 1500 rpm for 10 min, and the collected supernatant was analyzed using a multifunctional microplate reader (Tecan, Spark, Switzerland). The formula for calculating the hemolysis rate (%) was as follows:

Hemolysis rate (%) = (As − An) / (Ap − An) × 100 % (4)

Where As, An, and Ap represented the absorbance values of the sample, negative control group, and positive control group at 545 nm, respectively. The data obtained were subsequently analyzed for statistical significance using Prism software.

### 2.15 Detection of malondialdehyde (MDA) and glutathione (GSH) levels

MDA and GSH levels in BV2 cells were measured using an MDA assay kit (S0131, Bio-Tech, Shanghai, China) and a GSH activity assay kit (S0053, Bio-Tech, Shanghai, China), respectively. MDA and GSH levels were determined by measuring microplate fluorescence at 450 and 412 nm, respectively. These measurements were monitored using a multi-mode microplate reader, and the data obtained were subsequently analyzed for statistical significance using Prism software.

### 2.16 Determination of iron content

The total BV2 cellular iron content was determined using a tissue iron analysis kit (A039-2-1; Nanjing Jiancheng, China). In brief, in the presence of an acidic solution and a reducing agent, the ferrous iron in the tissue was reduced to ferrous iron, which combined with bipyridine to form a pink complex. Subsequently, the absorbance was measured with an enzyme marker. Absorbance was measured at 520 nm using a multi-mode microplate reader, and the data obtained were subsequently analyzed for statistical significance using Prism software.

### 2.17 Confocal microscopy of microglia

BV2 cells were incubated with fluorescent dyes diluted with DMEM, including MitoSOX Red Mitochondrial Superoxide Indicator (40778ES50, Yeasen), JC-1 (40705ES03, Yeasen), FerroOrange (F374, Dojindo, Japan), and Hochest 33342 (Beyotime Biotechnology, Nanjing, China). The BV2 cells were observed using an Nikon AX NSPARC fluorescence microscope (Nikon AX NSPARC, Japan).

#### 2.17.1 Quercetin target gene prediction

Structural information regarding quercetin was obtained from PubChem (https://pubchem.ncbi.nlm.nih.gov/). Quercetin targets were predicted from target fishing databases, namely, TCMSP (http://tcmspw.com/tcmsp.php), CHEMBL (https://www.ebi.ac.uk/chembl), and PubChem (https://pubchem.ncbi.nlm.nih.gov/PubChem), SwissTargetPrediction (http://www.swisstargetprediction.ch/), Pharmmapper (http://www.lilab-ecust.cn/pharmmapper/), STITCH (http://stitch.embl.de), and SuperPred (http://prediction.charite.de/) (Table S2). The SCI and NP related genes were accessed from GeneCards (www.genecards.org/). The ferroptosis-related genes were accessed from FerrDB Database (http://www.zhounan.org/ferrdb). The OS-related genes were accessed from CTD (https://ctdbase.org/). Venny2.1 (https://bioinfogp.cnb.csic.es/tools/venny/) was used to identify targets of quercetin in SCI, NP, ferroptosis, and OS.

#### 2.17.2 Protein–protein interaction network

Common targets were input into the STRING database (https://string-db.org/), the minimum required interaction score was set to 0.9, and outliers were deleted. The Cytoscape 3.9.1 software was used to import protein–protein interaction (PPI) data, build a PPI network, and identify hub targets. The CytoHubba plugin was used to determine the degree of interaction between the nodes. All the nodes of the PPI are presented in degree values, with the higher values being closer to the core. We used the following four algorithms for analysis: MCC, DMNC, BottleNeck, and EcCentricity.

#### 2.17.3 Gene ontology and kyoto encyclopedia of genes and genomes pathway enrichment analysis

The DAVID bioinformatics resource (david.ncifcrf.gov) was used to perform gene ontology (GO) and Kyoto Encyclopedia of Genes and Genomes (KEGG) pathway analyses of common targets. GO and KEGG results were ranked by count and P-value <0.05. The top 10 pathways were visualized as bubble plots using SRplot (www.bioinformatics.com.cn).

#### 2.17.4 Molecular docking technology

Chemical structures were obtained from the PubChem platform (https://pubchem.ncbi.nlm.nih.gov/). The three-dimensional (3D) structures of the proteins were downloaded from the Protein Data Bank (PDB) database (www.rcsb.org). Target proteins were dehydrated and dephosphorylated using PyMOL software. AutodockTools 1.5.7 was used to dock the target protein with the ligand quercetin molecule, binding energy analysis was performed, and the output was obtained in the PDBQT format. Finally, the results of molecular docking were visualized using PyMOl software.

#### 2.17.5 Molecular dynamics simulations

Molecular dynamics (MD) simulations of the Nrf2 and quercetin complexes were performed using GROMACS 2020.6 software. The AMBER99SB force field and SPC water model were used; the system temperature was set to 300 K; and the simulation time was 100 ns. The conjugate gradient method was used in the energy-minimization stage, followed by the NVT energy balance to stabilize the system, after which the MD simulation was completed. The MD simulation data obtained were visualized using Qtgrace software 0.2.6.

### 2.18 Animal experiments

#### 2.18.1 Construction of the SCI model

All animal experiments were performed in accordance with the Guidelines for the Care and Use of Experimental Animals and approved by the Ethics Committee of the Third Affiliated Hospital of Southern Medical University and the Animal Experimental Committee of the Daoke Pharmaceutical Technology (Guangdong) Co., Ltd (Approval No: IACUC-DK-2024-04-10-01). Adult male C57BL/6 mice (18–20 g, n = 120) were randomly divided into six groups: (1) sham (20 mice), (2) SCI (20 mice), (3) Que (20 mice), (4) MS (20 mice), (5) MSQ (20 mice), and (6) PGB (20 mice). The mice were anesthetized with sodium pentobarbital (50 mg/kg, i.p.), and laminectomy was performed at the level of the 10th thoracic vertebra. The T9–T10 spinal cord segments were exposed, and the SCI model was established by tapping the T9 segment with an SCI hammer (parameters: injury speed, 1.5 m/s; injury depth, 0.2 mm; dwell time, 0.5 s; hammer head diameter, 1.3 mm). After surgery, the muscles and skin were sutured; the bladder was squeezed manually twice daily for 2 weeks. Mice in the Que group were intraperitoneally injected of quercetin 7.5 mg/kg every 12 h for 14 and 28 days respectively, with the dosage regimen based on the previous sthdy [39]. Mice in the PGB group were intraperitoneally injected with 30 mg/kg pregabalin daily for 14 and 28 days respectively, with the dosage regimen based on the previous sthdy [40]. Mice in the MS and MSQ groups were administered precursor solutions of MS hydrogel and MSQ hydrogel respectively to the site of spinal cord injury postoperatively, and precursor solutions were exposed to UV light for 10 s to form hydrogels before the wound was sutured.

Furthermore, the Basso Mouse Scale (BMS) was used to evaluate motor function before surgery and at 1, 3, 7, 14, and 28 days after surgery. On postoperative day 14, the mechanical paw withdrawal threshold (PWT) and thermal paw withdrawal latency (PWL) were assessed in the hind paws of mice from each group. PWT was determined using a set of calibrated Von Frey Anesthesiometer (KW-CT-1, KEW BASIS, China), and the PWL was measured using the Plantar Test (IITC390G).

#### 2.18.2 Catwalk gait analysis

The Runway Scan system (version 3.0) was used to assess locomotor recovery. In brief, gait analysis was performed in a quiet environment 2 weeks after the injury. BCamCapture software was used by a blinded observer to analyze the treatment effect. Movement information of the mice was collected separately, including the regularity index (%), average speed (mm/s), stance time of the hindlimbs (ms), and paw presure of the hindlimbs.

#### 2.18.3 In vivo degradation imaging of fluorescently labeled hydrogel

Fluorescently labeled hydrogel, conjugated with Alkene coupling hydrogel fluorescent dyes (EFL-DYE-UF-ENE-R, Engineering for Life, China), was injected into the site of spinal cord injury in anesthetized mice. After a 3-min incubation to allow for dye expression, in vivo fluorescence imaging was performed using a fluorescence imaging system. Images were captured at 0-, 5-, 10-, and 14-days post-injection to monitor the degradation of the hydrogel within the living mice.

#### 2.18.4 Histological staining

Sections from different groups (sham, SCI, Que, MS, and MSQ) were immersed twice in xylene at room temperature for 20 min each. The sections were then immersed in a graded ethanol series (100 %, 90 %, 80 %, and 70 %) and double-distilled water for 10 min. The sections were stained with H&E reagent according to the manufacturer's instructions. For Nissl staining, the sections were stained with cresyl violet at 56 °C for 1 h and then differentiated in Nissl differentiation solution for 2 min. The sections were rapidly dehydrated by immersion in the following solutions in sequence: 95 % alcohol for 2 s twice, 100 % alcohol for 2 s, 100 % alcohol for 1 min, and xylene for 1 min twice. The sections were then finally sealed with neutral gum. Images of the target areas were captured using an optical microscope (Olympus, Tokyo, Japan), and the data obtained were subsequently analyzed for statistical significance using ImageJ software and Prism software.

#### 2.18.5 Immunohistochemical staining

Paraffin sections were prepared using the same procedure as for H&E staining. The sections were blocked after antigen retrieval. The sections were incubated with primary antibodies against IL-10 (cat#38392, SAB, USA), NueN (cat#ab177487, abcam, USA), MAP2 (cat#AB5622, Merck, Germany), 5-HT (cat#S5545, sigma, USA), CD206 (cat# 24595, CST, USA) and CD86 (cat# 19589, CST, USA) which was followed by incubation with the secondary antibodies for 30 min and subsequent development with diaminobenzidine substrate. The sections were then finally sealed with neutral gum. Images of the target areas were captured using an optical microscope (Olympus, Tokyo, Japan) and analyzed using ImageJ software and Prism software.

#### 2.18.6 Transmission electron microscopy

The mice were perfused with 100 mL of PBS and 50 mL of 4 % glutaraldehyde solution. The spinal cord segments were immersed in 4 % glutaraldehyde solution for fixation. After a series of dehydration treatments, ultrathin sections of the tissue blocks were obtained and placed on copper grids. Tissue ultrastructure was observed using a transmission electron microscope (H-7650, HITACHI, Japan).

### 2.19 Immunofluorescence staining

Immunofluorescence staining was performed on tissue sections and treated cells. After permeabilization and blocking, the sections and cells were incubated with the following primary antibodies: Iba1 (Cat# ab178846, Abcam, USA), Slc7a11 (Cat# ab175186, Abcam, USA), Gpx4 (Cat# ab125066, Abcam, USA), and Nrf2 (55136F, Abmart, China). The sections were then stained with the secondary antibodies for 1 h. Transferase-specific nick-end labeling (Tunel) staining was performed on frozen tissue sections. The cell nuclei in the tissue sections were detected using 4′,6-diamidino-2-phenylindole (Dapi) blocking reagent (Cat# ab104139, Abcam, USA). The corresponding photos were observed with an upright fluorescence microscope (Olympus, Tokyo, Japan) and analyzed using ImageJ software.

### 2.20 Western blotting

The samples were lysed in lysis buffer (Beyotime Biotechnology, Nanjing, China) containing protease inhibitor cocktails. Nuclear protein extraction and BCA Protein Assay Kits (Beyotime Biotechnology) were used for extraction and to determine protein concentrations, respectively. The protein samples were transferred to polyvinylidenedifluoride (PVDF) membranes, sealed for 1 h, washed with TBST solution, and then incubated with beta-actin (Cat# ab181602, Abcam, USA), Histone H3 (cat# P30266, Abmart, China), Gpx4 (Cat# ab125066, Abcam, USA), Slc7a11 (Cat# ab175186, Abcam, USA), and Nrf2 (cat#T55136, Abmart, China). The membranes were incubated with primary antibodies at 4 °C overnight. The membranes were washed the next day and incubated with horseradish peroxidase-labeled goat anti-rabbit IgG (1:5000; ab205718, Abcam) and goat anti-mouse IgG (1:5000; ab150113, Abcam) at room temperature for 1 h. Protein detection was performed using Biodlight™ ECL Chemiluminescent HRP Substrate (High Sensitivity) (BLH01S100, Bio World, USA) and the TANON-5200 chemiluminescence imaging system (Tanon Technology, Beijing, China). ImageJ software and Prism software were used for result analysis.

### 2.21 Quantitative reverse transcription polymerase chain reaction (qRT-PCR)

Total RNA was extracted using Trizol reagent (BS258A; Beyotime, China), and reverse transcription was performed using the RevertAid RT Reverse Transcription Kit (R222-01, Vazyme Biotech, China), following the manufacturer's guidelines. PCR amplification was executed using the Cham Q Universal SYBR qPCR Master Mix kit (Q711-02, Vazyme Biotech, China). The amplification protocol included an initial phase at 95 °C for 1 min, followed by 40 cycles at 95 °C for 15 s, and 50 °C for 30 s. Primer sequences are detailed in Table S2 (Supporting Information). The data obtained were subsequently analyzed for statistical significance using ImageJ software and Prism software.

### 2.22 Statistical analysis

All data are presented as mean ± standard error of the mean. Statistical significance was set at p < 0.05. One-way analysis of variance (ANOVA) with Tukey's or Dunnett's multiple-comparison post-hoc test was used for comparisons among multiple groups, and a two-tailed *t*-test was used for comparisons between two groups. A two-way ANOVA with a Bonferroni post-hoc test was used to assess the BMS scores. Electronic laboratory notebooks were excluded from this study.

## 3 Results and discussions

### 3.1 Preparation and characterization of MSQ hydrogel with synergistic protective effect on ROS homeostasis

**Fig. 1:**
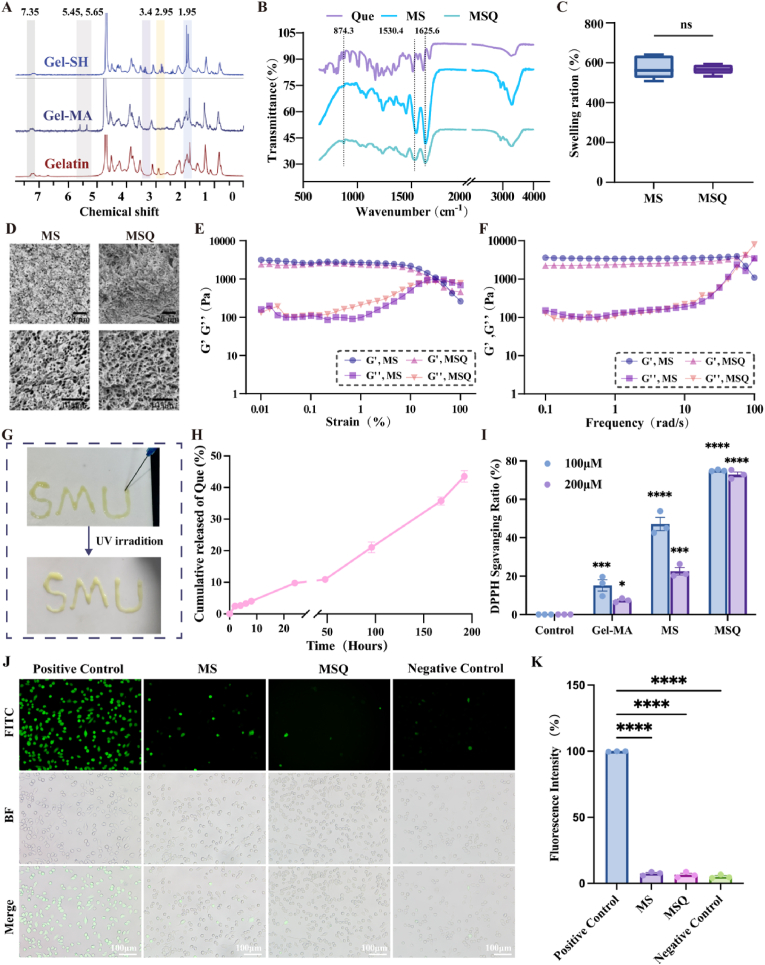
**Preparation and characterization of MSQ and ROS scavenging of MSQ hydrogel** (A)Nuclear magnetic resonance images of Galtin, Gel-SH, and Gel-MA. (B) FTIR spectra of quercetin, MS hydrogel, and MSQ hydrogel. (C)Swelling ratio of MS hydrogel and MSQ hydrogel. (n = 3) (D) SEM images of MS hydrogel and MSQ hydrogel, scale bar = 20μm/10 μm. (E, F) Rheological behaviors of MS hydrogel and MSQ hydrogel. (G) Photograph showing the injectability of the MSQ hydrogel. (H) Release curve of quercetin from MSQ hydrogel. (n = 3) (I) DPPH scavenging ratio of Gel-MA, MS hydrogel and MSQ hydrogel, compared with the control group, ∗P < 0.05, ∗∗∗P < 0.001, ∗∗∗∗P < 0.0001. (n = 3) (J, K) DCFH-DA assay for ROS scavenging capacity of MS hydrogel and MSQ hydrogel in BV2 cells in vitro, scale bar = 25 μm, compared with the positive control group, ∗∗∗∗P < 0.0001. (n = 3).

The SCI microenvironment is full of ROS and inflammatory cells, mainly microglia, which limit spinal cord repair in addition to causing NP [[41], [42], [43]]. The reduction of excess ROS and the phenotypic transformation of microglia are vital for alleviating NP and promoting neuronic repair [44,45]. Gelatin-based hydrogels are widely used as drug-delivery platforms due to their excellent biocompatibility, biodegradability, and low immunogenicity [46]. However, these hydrogels are easily influenced by stress, proteases, and ROS, resulting in uncontrollable drug release, various side effects, attenuated evenly deactivated bioactive or pharmacological effect. Herein, the injectable protective MSQ hydrogel composed of Gel-SH, Gel-MA, and quercetin was designed and prepared for the treatment of SCI, and the goal of loading quercetin was to control ferroptosis of microglia to reduce NP and promote spinal cord repair. Firstly, ^1^H NMR spectroscopy was performed to study the chemical structures of Gel-SH and Gel-MA. As seen from the ^1^H NMR spectra in Fig. 1A, GelSH that synthesized by carbodiimide method showed a characteristic peak at 3.4 ppm that was assigned to the protons of the methyne group of the NAC, and the peak at 1.95 ppm was assigned to the protons of the methylene group of the grafting NAC residual, while the peak at 2.95 ppm was assigned to lysine methylene of gelatin. The content of thiol groups was 212.5 μmol/g according to Ellman's method (Fig. S1). Additionally, as seen from the 1H NMR spectra of Gel-MA, the characteristic proton peaks were identified at 5.45 ppm and 5.65 ppm, which were associated with the protons on the carbon-carbon double bond of the side chain. Therefore, the result demonstrated that the methacrylic acid group was effectively incorporated into the gelatin molecular chain, and the degree of institution of methacrylic acid group was ∼38.7 %. The FTIR spectra of quercetin, MS hydrogel and MSQ hydrogel were performed (Fig. 1B). Compared to MS hydrogel, MSQ hydrogel encapsulated with quercetin showed a new band at 874.3 cm^−1^ attributed to the shift of the characteristic band at 882.5 cm^−1^ of quercetin, indicating the existence of quercetin. Additionally, the band 1625.6 cm^−1^ corresponded to the C=O stretching vibrations of the amide bond, while the band at 1530.4 cm^−1^ corresponded to the stretching vibrations of the amide bond.

The swelling analysis showed that the ESR of MS was 558.16 ± 27.36 %, which was close to the equilibrium swelling rate of MSQ (529.59 ± 18.62 %), and the ESR values of the two groups were not statistically significant difference (Fig. 1C, Fig. S2). These results indicated that the incorporation of quercetin did not greatly influence the crosslinking network based on Gel-SH and Gel-MA forming hydrogels via click chemistry. SEM analysis revealed that both MS hydrogel and MSQ hydrogel exhibited a substantial number of three-dimensional densely micro - and nano-porous structures (Fig. 1D). The average pore diameter of MS hydrogel and MSQ hydrogel were approximately 1.46 μm and 1.49 μm respectively (Fig. S3). These structural features shield ROS from surrounding tissue, and facilitated the sustained release of quercetin, effectively preventing an initial burst release. To evaluate the gelation time, we have monitored the rheological behaviors of MSQ hydrogel with different crosslinking time, as seen from Fig. S4, the storage modulus (G′) that represent the “solid-like” properties increased with the UV crosslinking time increasing (15 W/m^2^, 365 nm), and when the crosslinking time was 10s, the values of G′ was a little higher compare to the crosslinking time of sample was 6s, therefore, it indicated the hydrogels was completely crosslinked to form hydrogel after 10 s UV crosslinking. As shown in Fig. 1G and Fig. S5, the precursor solution of MSQ hydrogel was injected and then photocured to form hydrogel, and it was identified with shear thinning properties so that it was easy to administrate to the target area (Fig. S5). The rheological behaviors of the prepared hydrogels were also measured. At first, the storage modulus G′ was higher than the loss modulus G″, so both of MS and MSQ hydrogels showed solid-like behaviors. With shear strain and angular frequency increasing, the storage modulus G′ increased, finally decreased, indicating the disruption of MS and MSQ hydrogel structure (Fig. 1E and F). Notably, the moduli of the two hydrogel groups were nearly identical. It further indicated that the addition of quercetin did not affect the crosslinking network. The quercetin release profile was shown in Fig. 1H, it showed a low release rate and minimal total release within the first 24 h that consistent with the densely small pore sized microstructure features observed by SEM (Fig. 1D, Fig. S3), which effectively controlled the release of quercetin from MSQ hydrogel. Subsequently, the release rate and cumulative release amount linearly increased after 24 h, probably the most important reason was that the MSQ hydrogel was degraded to release quercetin (Fig. 1H). The results of the drug-release test indicated that quercetin was gradually released from the MSQ hydrogel at first due to its densely small pore sized microstructure, and then the release of quercetin was mostly influenced by the degradation of MSQ hydrogels, resulting the antioxidant properties of MSQ hydrogel being enhanced with the addition of quercetin (Fig. 1I–K).

The DPPH assay results demonstrated that the Gel-MA, MS, and MSQ hydrogel exhibited scavenging ability at both concentrations of 100 μM and 200 μM (Fig. 1I, Fig. S6). However, DPPH scavenging rates of the MS and MSQ groups were significantly higher than those of the Gel-MA group, and the MSQ group showed the highest DPPH-scavenging rate due to the synergy effects of reductive thiol group and quercetin, even the DPPH concentration increased from 100 μM to 200 μM. The superoxide anion (·O2^-^) scavenging ability was evaluated by pyrogallol autoxidation test, as shown in Fig. S7, the ·O2^-^ inhibition rate of MSQ hydrogel showed the highest of all, indicating that the best antioxidation of MSQ hydrogel due to the synergistic antioxidative effect between the thiol group and quercetin. Fig. 1J was the results of intracellular total ROS active assay. Brightfield observations confirmed the cells remained viable after treatment. However, the positive control showed a substantial amount of ROS in BV2 cells. While the BV2 cells cultured with MS and MSQ showed a significant reduction in ROS levels, and the MSQ group showed the most pronounced reduction. Quantitative analysis of fluorescence intensity was further used to scavenge the ROS that generated by oxidative stress in the treated cells (Fig. 1K). It showed that the fluorescence intensities in cells cultured with MS and MSQ were lower than that in the positive control group, while the fluorescence intensity in cells cultured with MSQ was close to the negative control groups, indicating effective removal of the generated excess ROS to maintaining intracellular ROS homeostasis. These intracellular ROS measurements were consistent with the results obtained from the DPPH assay. In conclusion, these results substantiated the ROS-scavenging capability of the MSQ hydrogel and its efficacy in maintaining intracellular ROS homeostasis that benefit for pretect the local cells amd tissues.

Combining the densely micro- and nano-porous morphologies structures, controlled release of quercetin, and synergistic antioxidant effects, a hypothesis of synergistic protective effect was proposed, which revealed that the reductive thiol groups synergistic with quercetin effectively scavenged the surrounding ROS to maintaining intracellular ROS homeostasis, therefore a synergistic protective barrier was developed, which protect the local cells including microglia and neurons, preserved the biological activity and efficacy of quercetin in targeting proteins in microglia.

### 3.2 In vitro biodegradability and biocompatibility of MSQ hydrogel

**Fig. 2:**
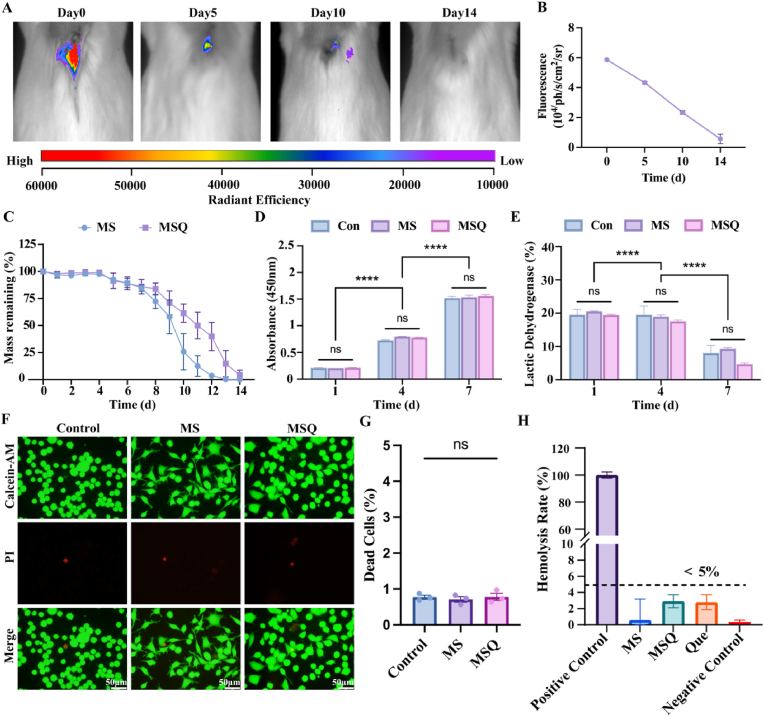
**In vitro and in vivo biocompatibility of MSQ hydrogel** (A) In vivo imaging of MSQ hydrogel implanted in mice, with images taken at 0-, 5-, 10-, and 14-days post-implantation. (B) The changes of the mean fluorescence intensity over time of MSQ hydrogel in vivo (n = 3). (C) In vitro degradation curves of MS and MSQ hydrogel. (D) Cell proliferation of BV2 cells treated with MS and MSQ hydrogel, assessed by CCK-8 assay (n = 3). (E) LDH release from BV2 cells treated with MS and MSQ hydrogel. (F, G) Live/dead staining of BV2 cells co-cultured with MS and MSQ hydrogel (scale bar = 50 μm) and corresponding statistical graphs. (n = 3) (H) Hemocompatibility of quercetin, MS and MSQ hydrogel. (n = 3).

Biodegradability is crucial for the development of biomaterials. In this study, the degradation behavior of the samples was assessed by weighing in vitro and also monitored by in vivo subcutaneous implantation of red fluorescent-labeled hydrogels. In vivo imaging of the red-fluorescent-labeled MSQ hydrogel demonstrates the progressive attenuation of the red fluorescence over time (Fig. 2A and B). By the 14th day, the fluorescence had completely dissipated, indicating that the subcutaneously implanted MSQ hydrogel had undergone complete degradation.it was consistence with the in vitro degradation of the hydrogel (Fig. 2C). The MSQ hydrogel sample initially exhibited a slow degradation rate, which subsequently increased over time. In comparison with the MS hydrogel, the MSQ hydrogel degraded at a slower pace, achieving complete degradation by day 14. These findings indicate that MSQ hydrogel possesses favorable biodegradability. The in vitro cytotoxic evaluation of MSQ hydrogel samples was assessed by the CCK-8 and the LDH assays. An increase in cell number was observed with culture time increasing (Fig. 2D), and no significant difference in proliferation rate was observed between the experimental and control groups. Fig. 2E illustrated a significant decrease in LDH levels as the culture time progressed, indicating high cellular activity and rapid growth. Live/dead staining assays were also used to assess the viability of the BV2 cells seeded in the hydrogels. In comparison with the control group, the MSQ hydrogel did not show cytotoxicity, as evidenced by the absence of significant differences in cell survival and proliferation rates. Quantitative statistical analysis of cell mortality revealed no statistically significant differences between the cell death rates in the sample and control groups, indicating that the samples did not induce substantial apoptosis (Fig. 2F and G). Additionally, as seen in Fig. 2H, the hemolysis rates for the MS, MSQ, and Que groups were all <5 %, indicating the good blood compatibility of the samples. These findings strongly indicated that the MSQ hydrogel exhibited excellent biocompatibility.

### 3.3 Analysis of the pharmacological targets of quercetin in post-SCI NP

**Fig. 3:**
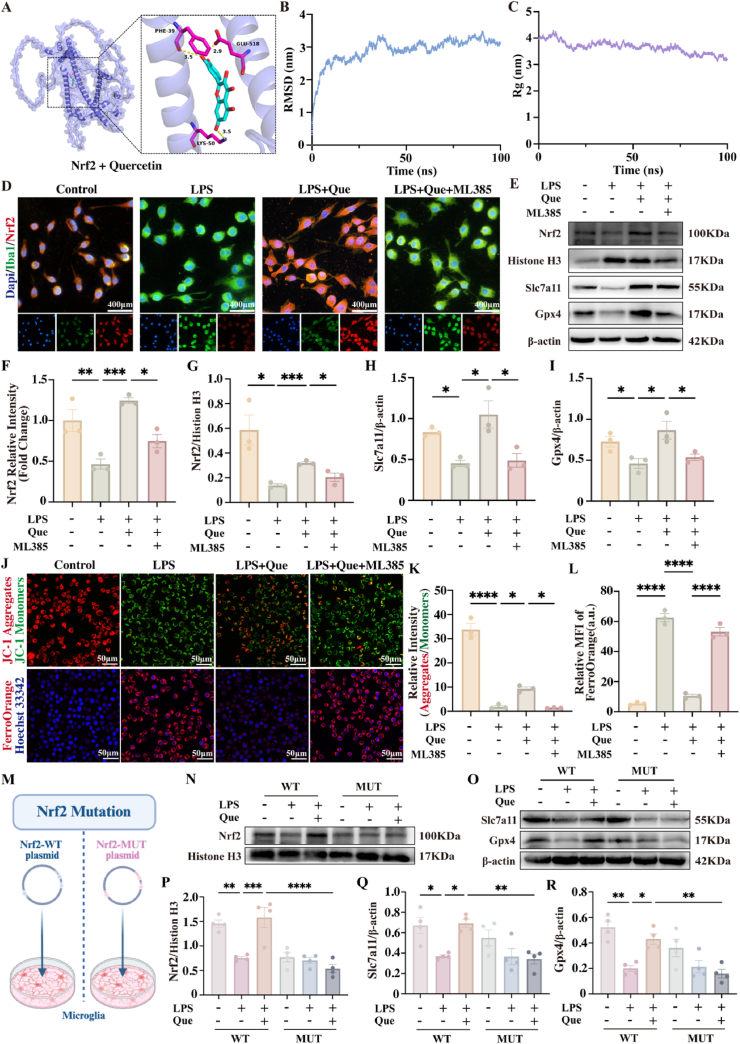
**Systems pharmacology analysis quercetin's microglial Nrf2 activation in post-SCI NP** (A) Molecular docking results of Nrf2 with quercetin. (B) Root Mean Square Deviation (RMSD) results of Nrf2 with quercetin. (C) Radius of Gyration (Rg) results of Nrf2 with quercetin. (D–F) Immunofluorescence staining images and statistical graphs of Nrf2 in BV2 cells, ∗P < 0.05, ∗∗P < 0.01, ∗∗∗P < 0.001, ∗∗∗∗P < 0.0001, scale bar = 400 μm (n = 3) (E, G-I) Western blot analysis statistical graphs of nuclear Nrf2 and total Slc7a11/Gpx4 expressions in BV2 cells ∗P < 0.05, ∗∗P < 0.01, ∗∗∗P < 0.001, ∗∗∗∗P < 0.0001. (n = 3) (J–L) Immunofluorescence staining images and statistical graphs of JC-1 and FerroOrange in BV2 cells, ∗P < 0.05, ∗∗∗∗P < 0.0001, scale bar = 50 μm (n = 3) (M) 2D interaction diagram of quercetin-Nrf2. (N, P) Western blot analysis statistical graphs of nuclear Nrf2 expression in BV2 cells, ∗∗P < 0.01, ∗∗∗P < 0.001, ∗∗∗∗P < 0.0001. (n = 4) (O, Q-R) Western blot analysis statistical graphs of total Slc7a11/Gpx4 expressions in BV2 cells, ∗P < 0.05, ∗∗P < 0.01. (n = 4).

To identify potential targets of the therapeutic effects of quercetin on post-SCI NP, we performed GO and KEGG enrichment analyses (Fig. S8A–B). Given the relationships of NP with oxidative stress and ferroptosis [47,48], we used an online platform to screen for targets related to quercetin (Table S3), NP, SCI, ferroptosis, and oxidative stress. A Venn diagram analysis revealed 22 common targets (Fig. S8C, Table S4), and we identified two key genes, Nrf2 and Ifng, using four algorithms (MCC, DMNC, BottleNeck and EcCentricity) (Fig. S8D–G). Molecular docking analysis revealed that quercetin binds strongly to Nrf2, with a binding energy of −8.3 kcal/mol (Fig. 3A, S10A), in comparison to a binding energy of −5.7 kcal/mol with Ifng (Fig. S9). Subsequently, we conducted a detailed MD simulation to explore the interaction of quercetin with Nrf2, which is essential for assessing conformational changes and the stability of ligand-protein complexes after docking. We used GROMACS to simulate the stability of the quercetin-Nrf2 complex, through a systematic analysis of multidimensional molecular dynamics indicators, the dynamic interaction mechanism of the Nrf2-quercetin complex was revealed. First, RMSD trajectory analysis indicated that the complex quickly reached a stable conformation after binding, with a significant conformational locking effect induced by quercetin (Fig. 3B). As shown in Fig. 3C, the Rg structural compactness evaluation showed a trend of overall structural densification, forming a spatially optimized stable topological framework. Combined analysis of solvent-accessible surface area (SASA) and binding free energy revealed that, during the early stages of binding, the complex underwent hydrophobic cavity reconstruction, forming an energetically optimized, stable binding mode (Fig. S10B–E). RMSF flexibility maps clarified the spatial distribution of rigid binding interfaces (low fluctuation regions) and functional flexible domains (moderate fluctuation regions) (Fig. S10F). The coordinated interactions between RMSD stability, Rg structural contraction, SASA energy optimization, and RMSF rigidity-flexibility distribution jointly form a “conformational locking-local adaptation” dynamic equilibrium mechanism, providing a molecular basis for the effective activation of the antioxidant signaling pathway. These findings provided further evidence that the interaction between quercetin and the target protein Nrf2 not only demonstrates high affinity, but also maintains stability under physiological conditions.

The Nrf2 pathway is vital for reducing lipid peroxidation and preventing ferroptosis by maintaining the redox balance and activating genes such as Gpx4 and Slc7a11, highlighting its role in cellular protection [49,50]. To investigate how quercetin inhibits ferroptosis by boosting Nrf2 expression in microglia and activating the Slc7a11/Gpx4 pathway, an in vitro model was constructed using LPS-stimulated BV2 cells that treated with quercetin and the Nrf2 inhibitor ML385 [51]. Immunofluorescence and nuclear fractionation-coupled Western blot analyses revealed that LPS decreased Nrf2 expression in BV2 cells, whereas quercetin increased it, and facilitates its translocation from the cytoplasm to the nucleus. However, ML385 effectively inhibited quercetin-induced Nrf2 upregulation and reduced its nuclear translocation (Fig. 3D–G). In parallel, we examined the expression of Nrf2 downstream targets involved in the ferroptosis pathway, Slc7a11 and Gpx4. In the LPS group, the protein levels of these targets were markedly diminished, signifying the occurrence of ferroptosis in BV2 cells. Conversely, quercetin treatment resulted in a significant upregulation of these proteins, while ML385 effectively suppressed the phenomenon (Fig. 3E–G-I, Fig. S11A–D). Collectively, these results suggested that quercetin modulate ferroptosis through the Nrf2/Slc7a11/Gpx4 pathway. To systematically evaluate oxidative stress and ferroptosis in BV2 cells, we demonstrated that quercetin effectively reversed LPS-induced glutathione (GSH) depletion and significantly suppressed the abnormal accumulation of malondialdehyde (MDA) and ferrous ions (Fe^2+^) (Fig. S12A–C). These protective effects were completely abolished by the Nrf2 inhibitor ML385. Mitochondrial functional analysis revealed that quercetin markedly inhibited LPS-triggered mitochondrial superoxide (MitoSOX) overproduction, an effect similarly blocked by ML385 (Fig. S13A–B). JC-1 staining further indicated mitochondrial membrane potential depolarization in both LPS and LPS + Quercetin + ML385 co-treated groups (Fig. 3J and K). Combined with FerroOrange iron-specific staining results (Fig. 3J–L), this study confirmed that quercetin inhibits ferroptosis in BV2 cells by upregulating Nrf2 expression. Furthermore, the qPCR analysis demonstrated that LPS treatment significantly upregulated inflammatory factors (TNFα, IL-1β, and IL-6) in BV2 microglial cells (Fig. S14A–C). Quercetin effectively attenuated this inflammatory response while upregulating anti-inflammatory M2 polarization markers (Arg1, IL-4, and IL-10), and these modulatory effects were reversed by ML385 co-treatment (Fig. S14D–E). In molecular docking studies, quercetin was found to form hydrogen bond interactions with Nrf2 at three critical residues: PHE39, LYS50, and GLU518. To investigate the binding mechanism of quercetin to Nrf2, we transfected BV2 cells with either wild-type Nrf2 (Nrf2-WT) or Nrf2 mutant (Nrf2-MUT, PHE39→ALA, LYS50→GLU, GLU518→ALA) plasmids (Fig. 3M). Nuclear lysate analysis demonstrated that the Nrf2-MUT abolished both Quercetin-induced Nrf2 expression and nuclear translocation (Fig. 3N–P). Furthermore, Nrf2-MUT transfection suppressed Slc7a11 and Gpx4 expression levels and attenuated Quercetin's protective effects against ferroptosis in BV2 cells (Fig. 3O–Q-R). Additionally, qPCR results indicated that Nrf2-MUT transfection inhibited Quercetin's effect in suppressing M1 polarization and promoting M2 polarization in BV2 cells (Fig. S15A–F). These findings confirmed that Quercetin facilitates Nrf2 dissociation and nuclear translocation by binding to the specific arginine residue, thereby promoting transcriptional regulation of Slc7a11 and Gpx4 expression, which mitigates LPS-induced ferroptosis and inflammation in BV2 cells.

### 3.4 MSQ hydrogel enhanced Nrf2 expression in BV2 cells to prevent ferroptosis and resist inflammation in vitro

**Fig. 4:**
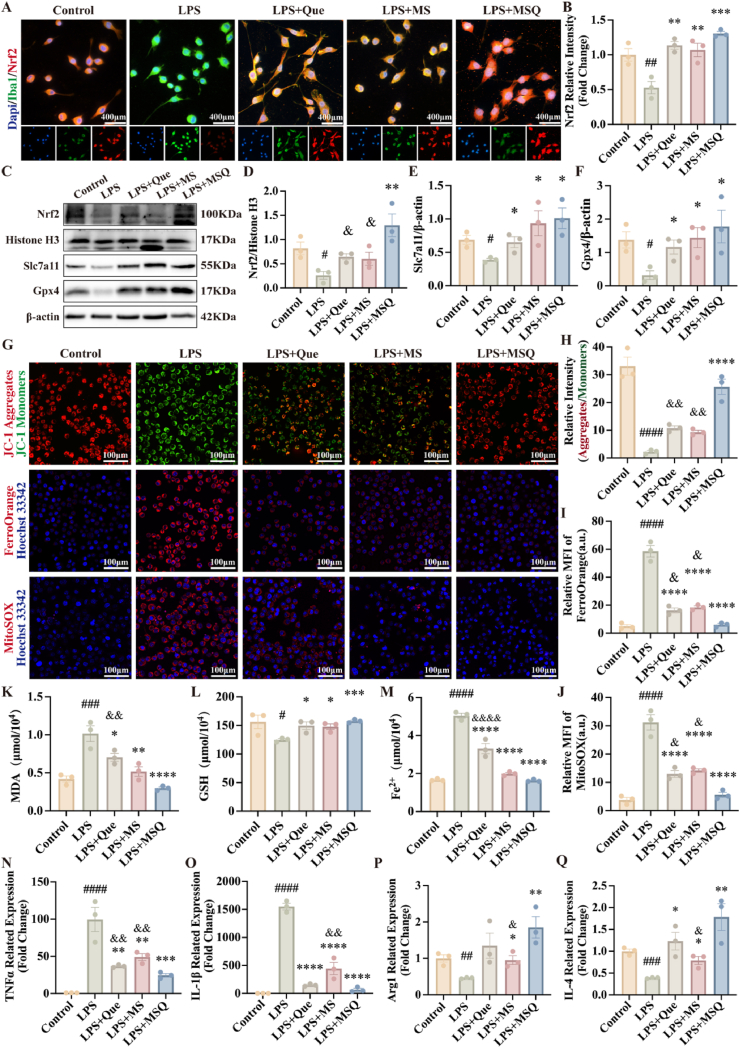
**The impact of MSQ hydrogel on ferroptosis and inflammatory response in BV2 cells** (A–B) Immunofluorescence staining images and statistical graphs of Nrf2 and Iba1 in BV2 cells, compared with Control group, ##P < 0.01, compared with LPS group, ∗P < 0.05, scale bar = 400 μm. (n = 3) (C-F) Western blot analysis and statistical graphs of nuclear Nrf2 and total Slc7a11 and Gpx4 expressions in BV2 cells, compared with Control group, #P < 0.05, compared with LPS group, ∗P < 0.05, ∗∗P < 0.01, compared with LPS + MSQ group, &P < 0.05. (n = 3) (G–J) Immunofluorescence staining images and statistical graphs of JC-1, FerroOrange and MitoSOX in BV2 cells, compared with Control group, ####P < 0.0001, compared with LPS group, ∗∗∗∗P < 0.0001, compared with LPS + MSQ group, &<0.05, &&P < 0.01, scale bar = 100 μm. (n = 3) (K–M) Determination of MDA, GSH, and Fe^2+^ levels in microglial cells, compared with Control group, #P < 0.05, ###P < 0.001, ####P < 0.0001, compared with LPS group, ∗P < 0.05, ∗∗P < 0.01, ∗∗∗P < 0.001, ∗∗∗∗P < 0.0001, compared with LPS + MSQ group, &&P < 0.01, &&&&P < 0.0001. (n = 3) (N–Q) qPCR analysis of inflammatory factors expressions (TNFα and IL-1β) and M2 factors expressions (Arg1 and IL-4) in BV2 cells, compared with Control group, ##P < 0.01, ###P < 0.001, ####P < 0.0001, compared with LPS group, ∗P < 0.05, ∗∗P < 0.01, ∗∗∗P < 0.001, ∗∗∗∗P < 0.0001, compared with LPS + MSQ group, &P < 0.05, &&P < 0.01. (n = 3).

To assess the effect of MSQ hydrogel on reducing microglial ferroptosis and inflammation, BV2 cells stimulated with LPS were treated with quercetin, MS hydrogel extract, and MSQ hydrogel extract in separate in vitro experiments. As seen from the results of immunofluorescence staining (Fig. 4A and B, S16A-D) and Western blot analyses (Fig. 4C–F), MSQ hydrogel significantly upregulation of nuclear Nrf2 expression and promotes its nuclear translocation, and the expression levels of Slc7a11 and Gpx4 were up regulated, and the activating efficacy of Slc7a11/Gpx4 pathway was greatest than the quercetin and MS groups. Mitochondrial functional analysis using JC-1 and MitoSOX probes in BV2 cells further demonstrated that MSQ hydrogel effectively restored mitochondrial homeostasis (Fig. 4G, H, J), concomitant with reduced Fe^2+^ accumulation (Fig. 4G–I, M) and MDA content, alongside enhanced glutathione (GSH) biosynthesis (Fig. 4K and L). These findings similarly indicated that MSQ hydrogel can effectively inhibit ferroptosis in BV2 cells (Fig. 4K–M) compared to other groups. The results proved that MSQ hydrogel could relief the oxidavie stress that induce microglial ferroptosis by the enhanced antioxidation. Concurrently, qPCR analyses also demonstrated that MSQ hydrogel effectively suppresses the expression of TNFα, IL-1β, and IL-6 (Fig. 4N–O, S17A), alongside increase M2 polarization markers (Arg1, IL-4, IL-10) (Fig. 4P–Q, S17B), indicating that MSQ hydrogel can synergize with quercetin to inhibit inflammatory responses in BV2 cells. Overall, these findings support the potential use of MSQ hydrogel to treat post-SCI NP by inhibiting microglia ferroptosis and inflammation which mostly induced by the abundant of ROS in the SCI microenvironment.

### 3.5 The therapeutic effects of MSQ hydrogel through the Nrf2/Slc7a11/Gpx4 pathway

**Fig. 5:**
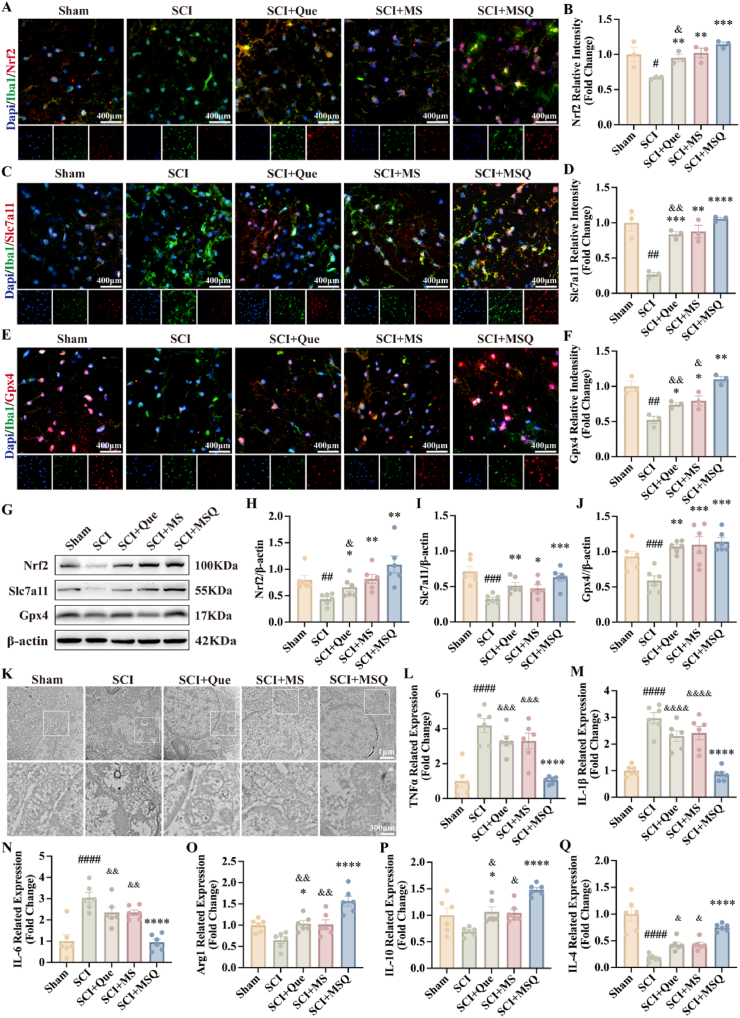
**The Impact of MSQ hydrogel on ferroptosis and neuroinflammation in microglia post-SCI NP** (A–F) Immunofluorescence staining images and statistical graphs of Nrf2, Slc7a11, Gpx4 and Iba1 in the spinal cord of mice after SCI, compared with Sham group, #P < 0.05, ##P < 0.01, compared with SCI group, ∗P < 0.05, ∗∗P < 0.01, ∗∗∗P < 0.001, ∗∗∗∗P < 0.0001, compared with MSQ group, &P < 0.05, &&P < 0.01, scale bar = 400 μm. (n = 6) (G–J) Western blot analysis and statistical graphs of Nrf2, Slc7a11, and Gpx4 expression in the spinal cord of mice after SCI, compared with Sham group, ##P < 0.01, ###P < 0.001, compared with SCI group, ∗P < 0.05, ∗∗P < 0.01, ∗∗∗P < 0.001, compared with MSQ group, &P < 0.05. (n = 6) (K) Transmission electron microscopy observation of mitochondrial morphology in microglia; scale bar = 1 μm/300 nm (L–Q) qPCR analysis of inflammatory factors expressions (TNFα, IL-1β, and IL-6) and M2 factors expressions (Arg1, IL-4, and IL-10) in the spinal cord of mice after SCI, compared with Sham group, ####P < 0.0001, compared with SCI group, ∗P < 0.05, ∗∗∗∗P < 0.0001, compared with MSQ group, &P < 0.05, &&P < 0.01, &&&P < 0.001, &&&&P < 0.0001. (n = 6).

Given the therapeutic effects of MSQ identified by the in vitro molecular biological experiments, the Nrf2/Slc7a11/Gpx4 pathway was further evaluated in vivo experiment at molecular level. After 14 days, spinal cord tissues were analyzed using immunofluorescence staining and Western blot. The results showed a marked decrease in Nrf2 protein expression after SCI, whereas the Que, MS, and MSQ groups exhibited a significant increase in Nrf2 expression, and MSQ groups exhibited highest expression level of Nrf2 (Fig. 5A–B, G-H). Subsequently, the expression levels of the Nrf2 downstream proteins, Slc7a11 and Gpx4, were assessed in the harvested SCI tissues. In comparison to Sham group, SCI group exhibited significantly decreased expression of Slc7a11 and Gpx4 (Fig. 5C–F, G, I-J), indicated the initiation of ferroptosis in the spinal cords of mice post-SCI. This observation aligns with previous research implicating ferroptosis in spinal cord injury [18], wherein the downregulation of these proteins is associated with the subsequent activation of microglial ferroptosis. Simultaneously, transmission electron microscopy was utilized to examine the ultrastructural modifications in microglia and their mitochondria post-SCI. Microglia in the sham group maintained a normal, intact morphology, whereas those in the SCI group exhibited a marked reduction in mitochondrial size, along with notable wrinkling of the inner mitochondrial membrane and rupture of the outer membrane. These observations further implied the occurrence of microglia ferroptosis post-SCI in mice. Transmission electron microscopy was used to study the post-SCI ultrastructural changes of microglia and the microglia mitochondria. In the sham group microglia maintained a normal intact structure, in contrast, microglia in the SCI group exhibited a marked reduction in mitochondrial size along with pronounced wrinkling of the inner membrane and rupturing of the outer membrane (Fig. 5K–S18). Nonetheless, in Que, MS, and MSQ groups, there was an upregulation in the expression of Slc7a11 and Gpx4, with the most pronounced increase observed in the MSQ group. Additionally, transmission electron microscopy revealed that the volume of microglia mitochondria in these groups closely resembled that of the Sham group, accompanied by a significant reduction in the degree of cristae invagination. Notably, the MSQ group exhibited the most substantial recovery of microglia mitochondrial morphology. These findings suggested that quercetin, MS hydrogel, and MSQ hydrogel are effective in mitigating ferroptosis in microglia, however the MSQ hydrogel exhibiting the most pronounced therapeutic efficacy due to its strong antioxiadation and higher drug delivery effects. The inflammatory response in the spinal cord after SCI in mice was evaluated using qPCR. The results showed that inflammatory cytokines TNFα, IL-1β, and IL-6 were significantly lower in the Que, MS, and MSQ groups than in the SCI group, with the MSQ group showed the most significant reduction, indicating the anti-inflammation of MSQ hydrogel (Fig. 5N and O S17A). Concurrent upregulation of M2 factors (Arg1, IL-4, IL-10) specifically in the MSQ group (Fig. 5P–Q, S17B). Immunohistochemical staining was further verified that the MSQ hydrogel can promote the inflammation microenvironment to anti-inflammation microenvironment, which showed the expression of CD86 decreased while the expression of CD206 increased (Fig. S19). These findings suggested that MSQ hydrogel inhibits ferroptosis in microglia through Nrf2/Slc7a11/Gpx4 pathway in vivo, consequently attenuating neuroinflammation and conferring protective effects on spinal cord tissues due to the synergistic protective effect and maintaining the intracellular redox homeostasis.

### 3.6 MSQ hydrogel promoted motor function recovery and alleviated post-SCI NP

**Fig. 6:**
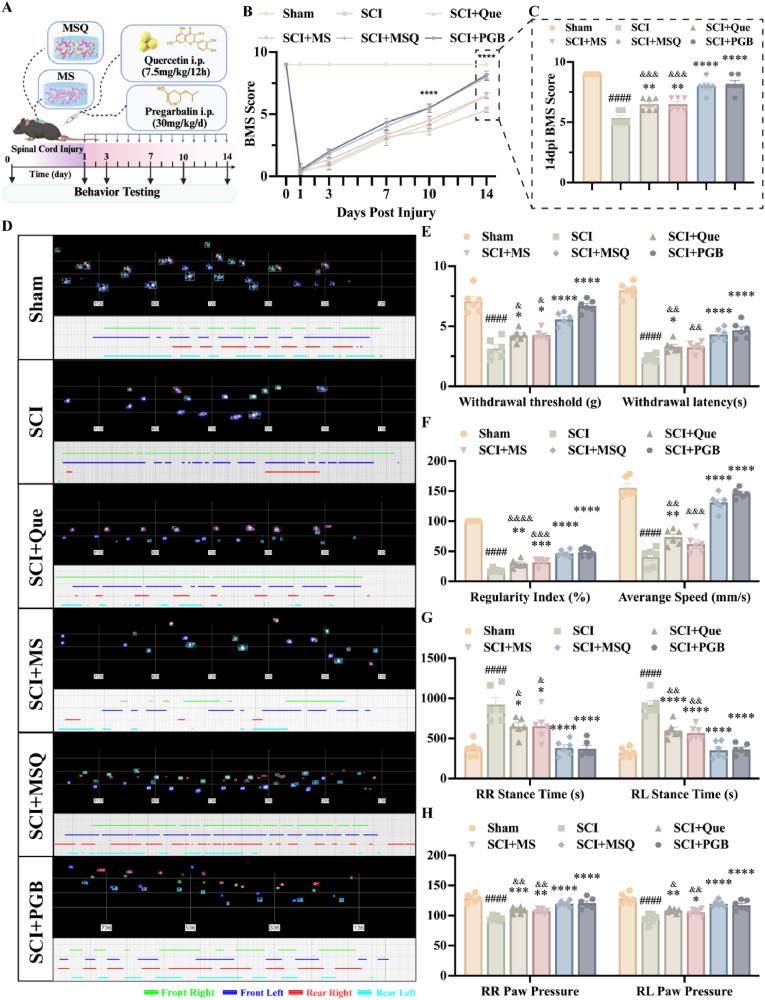
**The Impact of MSQ hydrogel on motor function and neuropathic pain in mice post-SCI** (A) Experimental timeline showing intervention and assessment windows. (B) BMS scores of mice in each group at 0-, 1-, 3-, 7-, 10-, and 14-days post-SCI, compared with the SCI group, ∗∗∗∗P < 0.0001. (n = 6) (C) BMS scores of mice in each group on day 14 post-SCI, compared with the Sham group, ####P < 0.0001, compared with the SCI group, ∗∗P < 0.01, ∗∗∗∗P < 0.0001, compared with the MSQ group, &P < 0.001. (n = 6) (D) CatWalk gait analysis of footprint shape and sequence in SCI mice at 14 days post-injury. (E) Mechanical pain threshold testing and thermal pain threshold testing of mice in each group on day 14 post-SCI, compared with the Sham group, ####P < 0.0001, compared with the SCI group, ∗P < 0.05, ∗∗∗∗P < 0.0001, compared with the MSQ group, &P < 0.05, &&P < 0.01. (n = 6) (F-H) Quantitative analysis of footprints, characterizing the interlimb coordination and hindlimb strength of mice. (F) Gait index (ratio of regular steps to total steps) and average speed (mm/s). (G) RR, right hindlimb, RL, left hindlimb, maximum average stance time (ms). (H) RR, right hindlimb, RL, left hindlimb, maximum average paw pressure. Compared with the Sham group, ####P < 0.0001, compared with the SCI group, ∗P < 0.05, ∗∗P < 0.01, ∗∗P < 0.001, ∗∗∗∗P < 0.0001, compared with the MSQ group, &P < 0.05, &&P < 0.01, &&&P < 0.001, &&&&P < 0.0001. (n = 6).

To comprehensively evaluate the therapeutic effects of MSQ hydrogel on post-SCI NP, the mouse SCI model was established with pregabalin serving as the positive control (Fig. 6A). After a 14-day observation period, motor function and pain indicators were evaluated using the Basso Mouse Scale (BMS) score, catwalk gait analysis, and assessments of mechanical and thermal pain sensitivity. As depicted in Fig. 6B, no significant differences in BMS scores were observed among the groups prior to the 10th day. However, starting from the 10th day, the BMS scores of MSQ group were significantly higher than those of SCI, Que, and MS groups. By the 14th day, the hindlimb muscle strength in the MSQ group demonstrated a marked recovery (Fig. 6C). The results of the catwalk analysis were shown in Fig. 6D, on day 14 post-SCI, the hindlimb footprints of mice in the sham group were distinct, indicating normal hindlimb function. In contrast, those in the SCI group exhibited significantly impaired hindlimb movement, resulting in indistinct footprints. This observation suggested that hind limb function had not recovered in the SCI group. Although hindlimb movements in MSQ groups were less pronounced than those in the sham group, their footprints showed best than those in SCI group, Que group, and MS group (Fig. 6D). Moreover, the recovery of hindlimb motor function was assessed through core parameters including gait regularity index, average movement speed, and stance time (Fig. 6F and G). The SCI group exhibited significant reductions in regularity index and movement speed, coupled with prolonged stance time, indicating incomplete neuromotor compensation. All treatment groups (quercetin, MS hydrogel, MSQ hydrogel, and pregabalin) demonstrated upward trends in these metrics, with the MSQ hydrogel and pregabalin groups showing the most pronounced improvements, confirming the efficacy of these interventions in promoting hindlimb motor functional reconstruction. Concurrent analysis of the maximum average paw pressure (Fig. 6H), a dual indicator of functional recovery and pain assessment, revealed characteristic declines in the SCI group. Notably, the MSQ hydrogel group exhibited values closest to the PBG group, further validating its synergistic therapeutic advantages in simultaneously addressing motor function restoration and neuropathic pain alleviation via multi-target regulation mechanisms. These results demonstrated that the MSQ hydrogel effectively improved these indices, thereby proving its healing effects.

To further investigate the therapeutic effects of MSQ hydrogel on post-SCI NP, the mechanical and thermal pain thresholds were assessed (Fig. 6E). By 14 days post-SCI, the mice of SCI group exhibited a significant reduction in both mechanical and thermal pain sensitivity with the lowest withdraw threshold and latency. However, the groups treated with quercetin, MS hydrogel, MSQ hydrogel and pregabalin showed improvements in these thresholds, and MSQ group and pregabalin demonstrated the most pronounced enhancement in pain sensitivity compared to Que and MS groups. This indicated that quercetin, MS hydrogel, and MSQ hydrogel have therapeutic effects on post-SCI NP, however MSQ hydrogel showed the best efficacy. Therefore, MSQ hydrogel had the potential to alleviate post-SCI NP. These findings suggested that MSQ hydrogel can effectively alleviate NP and promote the recovery of motor function in mice after SCI because of the synergistic protective effect and high drug delivery effects.

### 3.7 Antiinflammation and cell apoptosis resistance in vivo of MSQ hydrogel to enhance SCI recovery

**Fig. 7:**
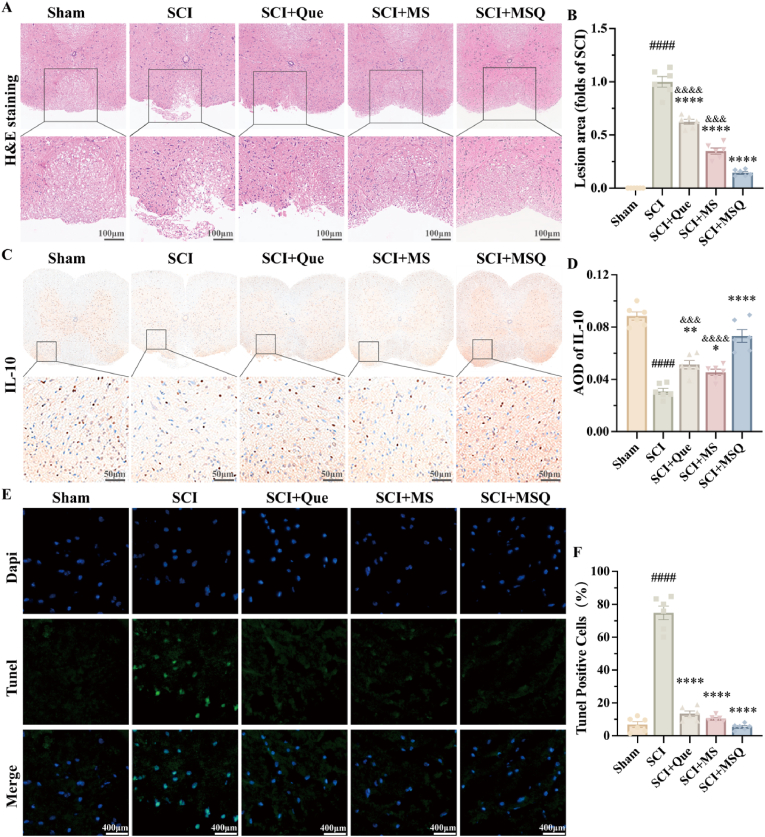
**The Impact of MSQ Hydrogel on Tissue Regeneration and Mitigation of Cellular Apoptosis Following SCI** (A–B) H&E staining of spinal cord tissues from mice in each group post-SCI (scale bar = 100 μm) and statistical charts of spinal cord injury area, compared with the Sham group, ####P < 0.0001; compared with the SCI group, ∗∗∗∗P < 0.0001; compared with the MSQ group, &&P < 0.01, &&&P < 0.001. (n = 6) (C–D) IL-10 staining of spinal cord tissues from mice in each group post-SCI (scale bar = 50 μm) and statistical charts, compared with the Sham group, ####P < 0.0001; compared with the SCI group, ∗P < 0.05, ∗∗P < 0.01, ∗∗∗∗P < 0.0001; compared with the MSQ group, &&&P < 0.001, &&&&P < 0.0001. (n = 6) (E–F) TUNEL staining of spinal cord tissues from mice in each group post-SCI (scale bar = 400 μm) and statistical charts, compared with the Sham group, ####P < 0.0001; compared with the SCI group, ∗∗∗∗P < 0.0001. (n = 6).

After SCI, localized neuron loss and cavity formation occur, while secondary injuries like ferroptosis and inflammation lead to glial scar hyperplasia, hindering axonal growth and worsening NP [52]. Filling the cavity in lesions is a crucial research focus. We performed H&E staining of paraffin-embedded mouse spinal cord sections to assess the severity of SCI across the experimental groups (Fig. 7A and B). The results indicated that quercetin, MS, and MSQ improved histopathological structures post-SCI, with MS hydrogel and MSQ hydrogel showing significantly better outcomes than quercetin alone. Immunohistochemical analysis also revealed a marked reduction in IL-10-positive cells after SCI (Fig. 7C and D). However, treatment with quercetin, MS, and MSQ resulted in a significant elevation in IL-10-positive cells, and the MSQ treatment group showed the most substantial increase, indicating MSQ hydrogel effectively attenuated the inflammatory response and showed anti-inflammation. TUNEL staining was employed to assess apoptosis in murine spinal cord cells after SCI modeling (Fig. 7E and F). The findings indicated that MSQ hydrogel demonstrated the greatest efficacy to mitigate apoptosis induced by SCI. Overall, these findings strongly suggest that MSQ hydrogel significantly reduces inflammation and cell death while protecting and aiding the recovery of damaged spinal cord tissue, due to the quercetin incorporated in MSQ hydrogel was diliveried effectively and showed high bioactive and pharmacological effect.

### 3.8 Regeneration of SCI tissues treated with MSQ hydrogel

**Fig. 8:**
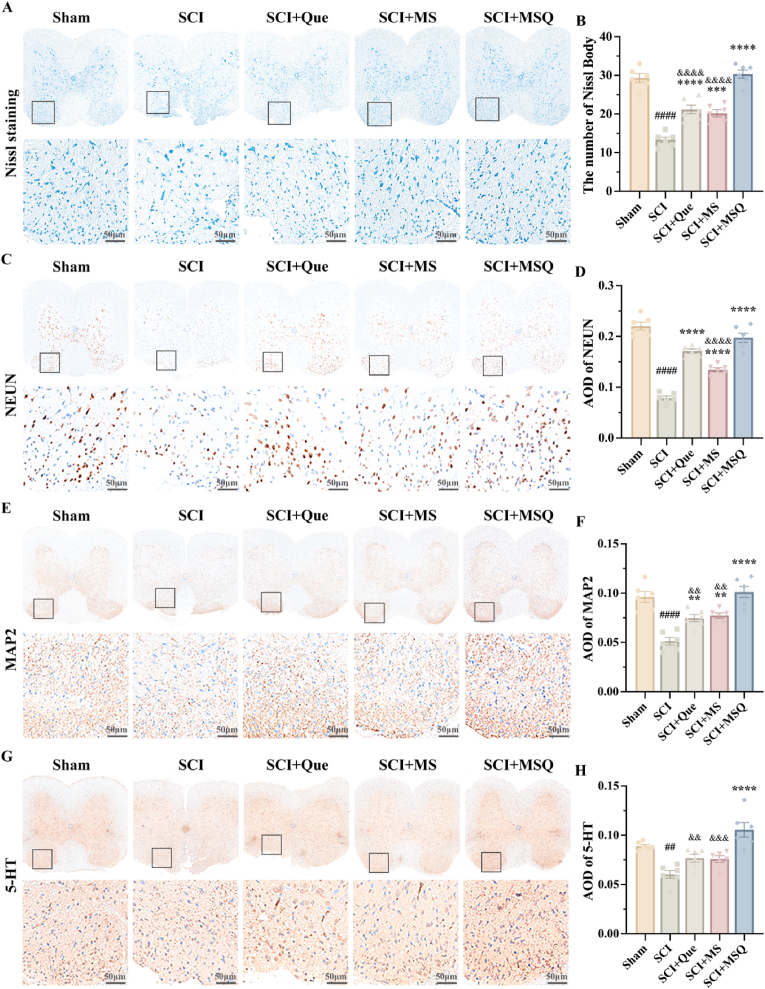
**MSQ hydrogel promotes neuron regeneration.** (A) Nissl staining of spinal cord tissues from mice in each group post-SCI (scale bar = 50 μm) and statistical charts of Nissl body counts, compared with the Sham group, ####P < 0.0001, compared with the SCI group, ∗∗∗P < 0.001, ∗∗∗∗P < 0.0001, compared with the MSQ group, &&&&P < 0.0001. (n = 6) (B-C) Immunohistochemical staining for NeuN, MAP2, and 5-HT in spinal cord tissues from mice in each group post-SCI (scale bar = 50 μm) and statistical charts of positive cell counts, compared with the Sham group, ##P < 0.01, ####P < 0.0001, compared with the SCI group, ∗∗P < 0.01, ∗∗∗∗P < 0.0001, compared with the MSQ group, &&P < 0.01, &&&P < 0.001, &&&&P < 0.0001. (n = 6).

After SCI, the normal neurons should be protected from apoptosis induced by excess ROS in neuroinflammation microenvironment, and neurotic repair is important for the rehabilitation of SCI patient. Herein, neuronal populations were evaluated using Nissl and NeuN staining techniques (Fig. 8A–D). It showed a significant reduction in the number of Nissl bodies and NeuN-positive cells post-injury. In comparison with the SCI group, the groups treated with quercetin, MS hydrogel, and MSQ hydrogel showed an increase in the number of Nissl bodies and NeuN-positive cells. Notably, MSQ hydrogel exhibited the most pronounced effect in enhancing the number of Nissl bodies and NeuN-positive cells. The results identified the synergistical protective effects of MSQ on the local tissue cells. To evaluate the restoration of neuronal architecture and dendritic arborization, as well as the functionality of the neurotransmitter system, the immunohistochemical staining of MAP2 for dendritic integrity and 5-HT for neurotransmission was assessed in the affected regions post-SCI (Fig. 8E–G). In comparison with the SCI group, the groups treated with quercetin, MS hydrogel, and MSQ hydrogel showed significant increments in the number of MAP2- and 5-HT-positive cells within the spinal cord, indicating the improvement of neuronal function. Notably, the most expression of MAP2 and 5-HT in MSQ hydrogel treatment group showed the most, which strongly indicated that MSQ hydrogel can effectively and significantly enhance spinal cord recovery following SCI due to the synergy protection effects.

### 3.9 Long-term therapeutic efficacy and systemic Safety evaluation of MSQ hydrogel in SCI

**Fig. 9:**
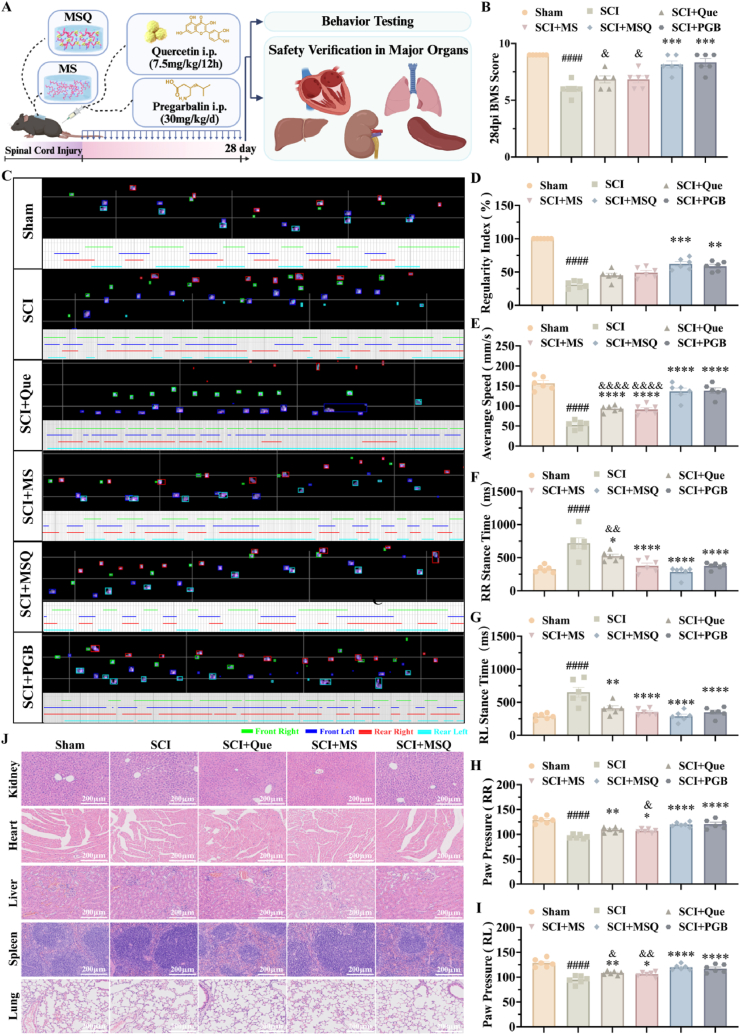
**MSQ hydrogel-mediated functional recovery and systemic biocompatibility at 28 days post-SCI** (A) Timeline illustration of the experimental design. (B) BMS scores of mice in each group on day 28 post-SCI, compared with the Sham group, ####P < 0.0001, compared with the SCI group, ∗∗P < 0.01, ∗∗∗∗P < 0.0001, compared with the MSQ group, &P < 0.05. (n = 6) (C-I) Catwalk gait analysis of mice in each group on day 28 post-SCI. (C) Description of footprint shape and sequence. (D–I) Quantitative analysis of footprints, characterizing the interlimb coordination and hindlimb strength of mice. (D) Gait index (ratio of regular steps to total steps). (E) Average speed (mm/s). (n = 6) (F, G) RR, right hindlimb, RL, left hindlimb, maximum average stance time (ms). (n = 6) (H, I) RR, right hindlimb, RL, left hindlimb, maximum average paw pressure. Compared with the Sham group, ####P < 0.0001, compared with the SCI group, ∗P < 0.05, ∗∗P < 0.01, ∗∗P < 0.001, ∗∗∗∗P < 0.0001, compared with the MSQ group, &P < 0.05, &&P < 0.01, &&&&P < 0.0001. (n = 6) (J) H&E staining images of mice major organs (kidney, heart, liver, spleen, lung) at day 28 post-SCI, scale bar = 200 μm. (n = 6).

To systematically evaluate the long-term therapeutic efficacy of MSQ hydrogel on spinal cord injury, functional assessments were conducted after 28 days post-injury using the Basso Mouse Scale and CatWalk gait analysis system (Fig. 9A). Comparative analysis between 14-day and 28-day timepoints revealed that the SCI group's hindlimb functional recovery rate progressively decelerated, ultimately demonstrating significantly lower BMS scores compared to all therapeutic intervention groups (quercetin, MS hydrogel, MSQ hydrogel, and pregabalin) at the terminal 28-day evaluation (Fig. 9B). This temporal resolution of therapeutic effects was further corroborated by CatWalk gait analysis, where the SCI group exhibited limited improvement in index regularity and average speed from baseline to 28 days, in stark contrast to the substantial enhancements observed in all treatment groups over the same period (Fig. 9C–E). Particularly noteworthy was the MSQ hydrogel group, which demonstrated accelerated recovery kinetics between 14 and 28 days, ultimately achieving functional parity with the clinically established pregabalin in gait coordination and load-bearing capacity parameters (Fig. 9F–I). Compared to quercetin and MS hydrogel, MSQ hydrogel was significantly improved hindlimb motor dysfunction indicating that MSQ hydrogel improved and increased the recover abilitis of SCI mice. Notably, MSQ hydrogel exhibited therapeutic equivalence to the clinically established drug pregabalin in core parameters including gait coordination and hindlimb load-bearing capacity, indicating its potential for clinical application in neural functional recover. In addition, H&E staining of the major organs (kidney, heart, liver, spleen, lung) of the mouse after treatment was conducted to evaluate the systemic toxic reactions (Fig. 9J), and all groups had no obvious tissue toxicity and obviated systemic infection, thus it verified the good biocompatibility of MSQ hydrogel. These findings confirmed that the MSQ hydrogel effectively alleviates neuropathic pain following spinal cord injury in murine models. The comprehensive data collectively demonstrate the therapeutic potential of this innovative hydrogel system for neural repair applications, supported by its demonstrated efficacy and favorable biosafety profile.

Hydrogels loading with Chinese medicine are being increasingly recognized as a promising strategy for the treatment of post-SCI NP [53,54]. Although previous research has indicated that the synergistic reduction of ROS and the inhibition of ferroptosis constitute effective strategies for mitigating neuroinflammation in the management of SCI, our findings demonstrated that treatment with MSQ effectively reduces excess ROS in microglia and inhibits cell ferroptosis by activating the Nrf2/Slc7a11/Gpx4 pathway, thereby mitigating the inflammatory response. Importantly, this study is the first to elucidate the intricate mechanisms underlying the therapeutic effects of this strategy in addressing NP during SCI treatment. These insights not only enhance the translational potential of our research, but also lay the groundwork for the development of broader therapeutic interventions targeting post-SCI NP and spinal cord repair.

## 4 Conclusions

In this study, we developed a MSQ hydrogel with synergistic anti-inflammatory and ROS-scavenging abilities to mitigate the advancement of post-SCI NP and promote neural repair. MSQ hydrogel effectively scavenged ROS due to its synergy effects with the sustained release of quercetin induced by biodegradation and showed a synergistic protective effect that protects the nerve cells, reduce microglia ferroptosis, and promote anti-inflammation phenotype of microglia. MSQ exhibited a significant therapeutic effect on pain scores and motor function repair, which was demonstrated by marked improvements in the BMS scores, catwalk analysis, mechanical pain threshold scores, and thermal pain threshold scores. Furthermore, at the molecular mechanism levels, MSQ hydrogel exerted its effects by inhibiting microglia ferroptosis and attenuating cellular inflammation via the Nrf2/Slc7a11/Gpx4 pathway. In conclusion, we developed an injectable protective MSQ hydrogel with antioxidation, anti-inflammation and microglia ferroptosis inhabitation. The underlying therapeutic mechanisms was elucidated thoroughly at multi-level from in vitro evaluation to molecular mechanism in vivo, which revealed the inhibition of spinal microglial ferroptosis and inflammatory response by activating the Nrf2/Slc7a11/Gpx4 pathway. This material-biology synergy ensures sustained pathway activation beyond conventional antioxidant therapies This study offers valuable insights into the development of biomaterials for treating post-SCI NP and promoting spinal cord repair.

## CRediT authorship contribution statement

**Lu Li:** Writing – review & editing, Writing – original draft, Resources, Investigation, Data curation. **Yu Cao:** Software, Methodology, Formal analysis. **Xiangsheng Zhang:** Validation, Investigation, Data curation. **Jiayi Guo:** Visualization, Methodology. **Ziqiang Lin:** Formal analysis. **Pengyu Zhou:** Methodology. **Chuyin Chen:** Validation. **Jiahao Chen:** Software. **Yike Liu:** Data curation. **Danzhi Luo:** Investigation. **Jiurong Chen:** Methodology. **Yingdong Deng:** Visualization. **Peng Sun:** Visualization, Supervision. **Zhiwen Zeng:** Writing – review & editing, Validation, Supervision. **Jun Zhou:** Writing – review & editing, Investigation, Funding acquisition, Conceptualization.

## Ethics approval and consent to participate

The animal experiments were approved by the Animal Experimental Committee of the Daoke Pharmaceutical Technology (Guangdong) Co., Ltd (Approval IACUC-DK-2024-04-10-01) and were conducted in compliance with the guidelines stipulated in the National Act on the Use of Experimental Animals (People's Republic of China).

## Declaration of competing interest

The authors assert that the research was carried out without any commercial or financial affiliations that could be perceived as a possible conflict of interest.

## Data Availability

Data will be made available on request.
