# Supplementary figures and images for: Injectable ROS homeostasis protective hydrogel inhibiting microglial ferroptosis through the Nrf2/Slc7a11/Gpx4 to alleviate neuropathic pain and promote spinal cord injury repair
Source: Redox Biol. 2025 Aug 8;86:103816. doi: 10.1016/j.redox.2025.103816 (PMC12359189; doi:10.1016/j.redox.2025.103816)

**Figure 3E**

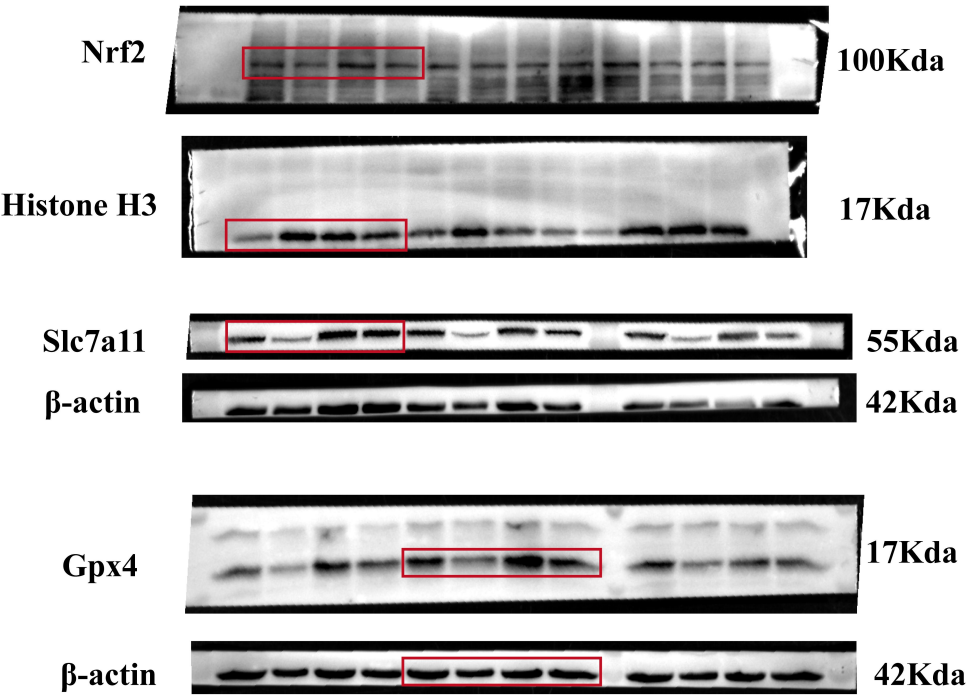

**Figure 3N**

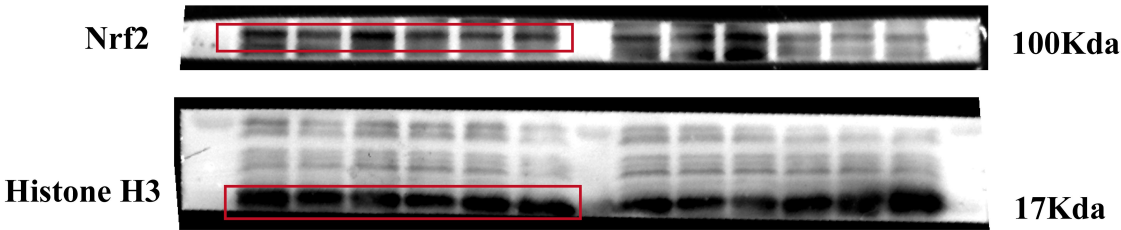

**Figure 3O**

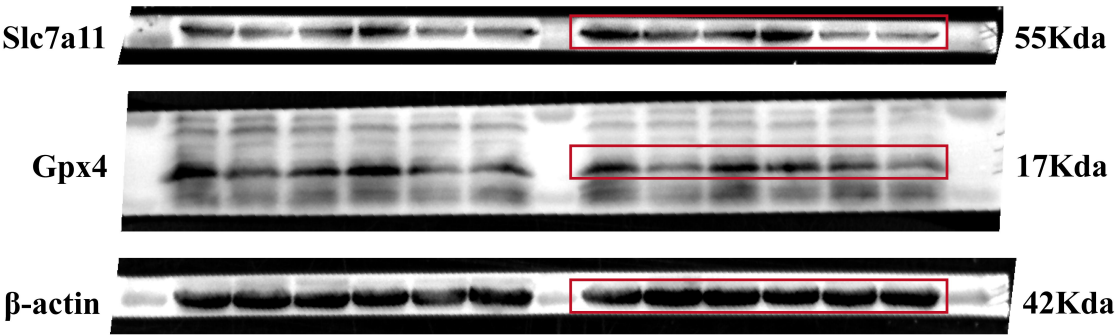

**Figure 4C**

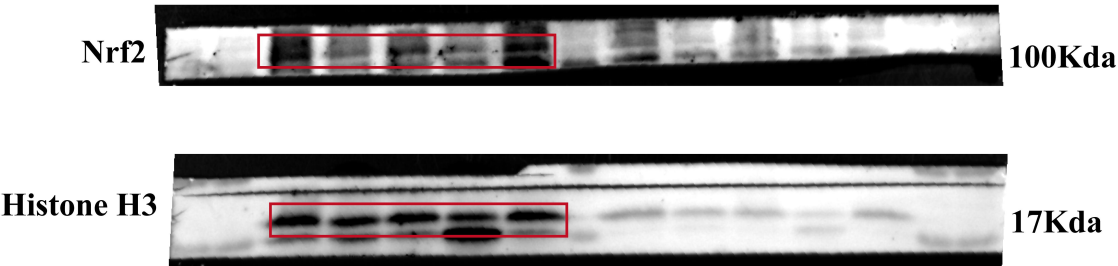

Figure 4C

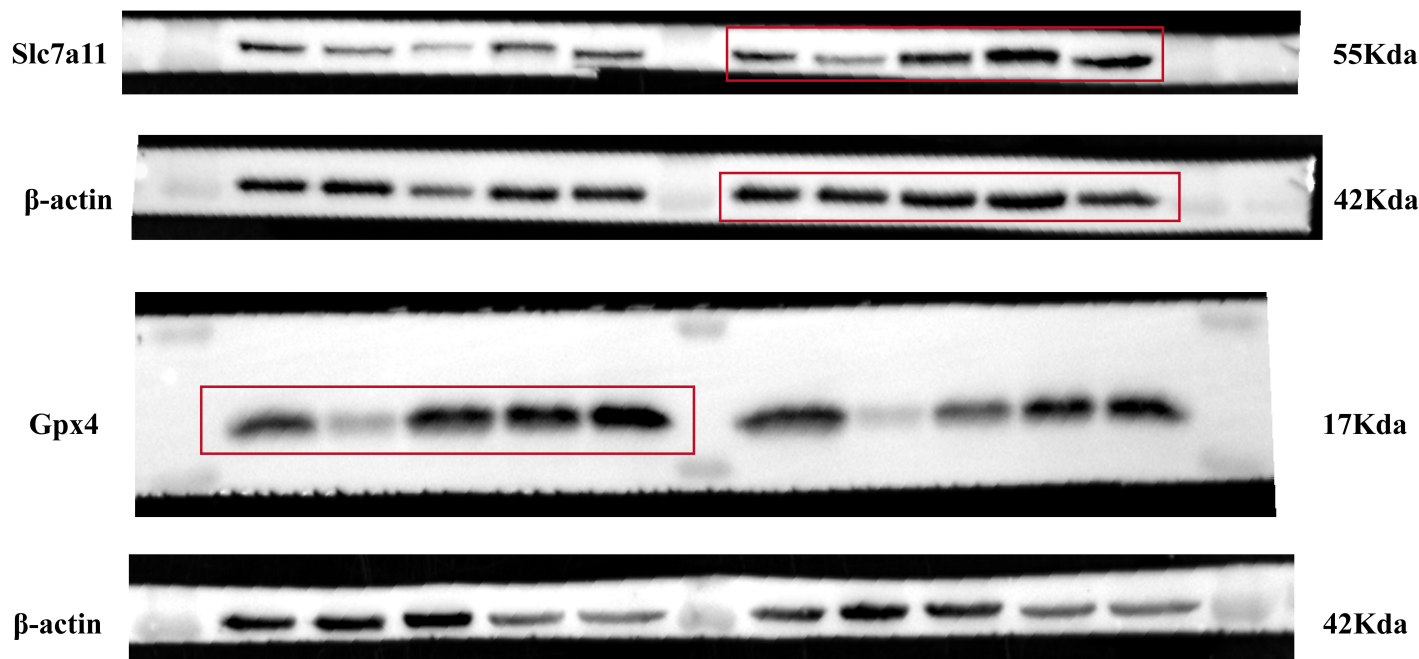

Figure 5G

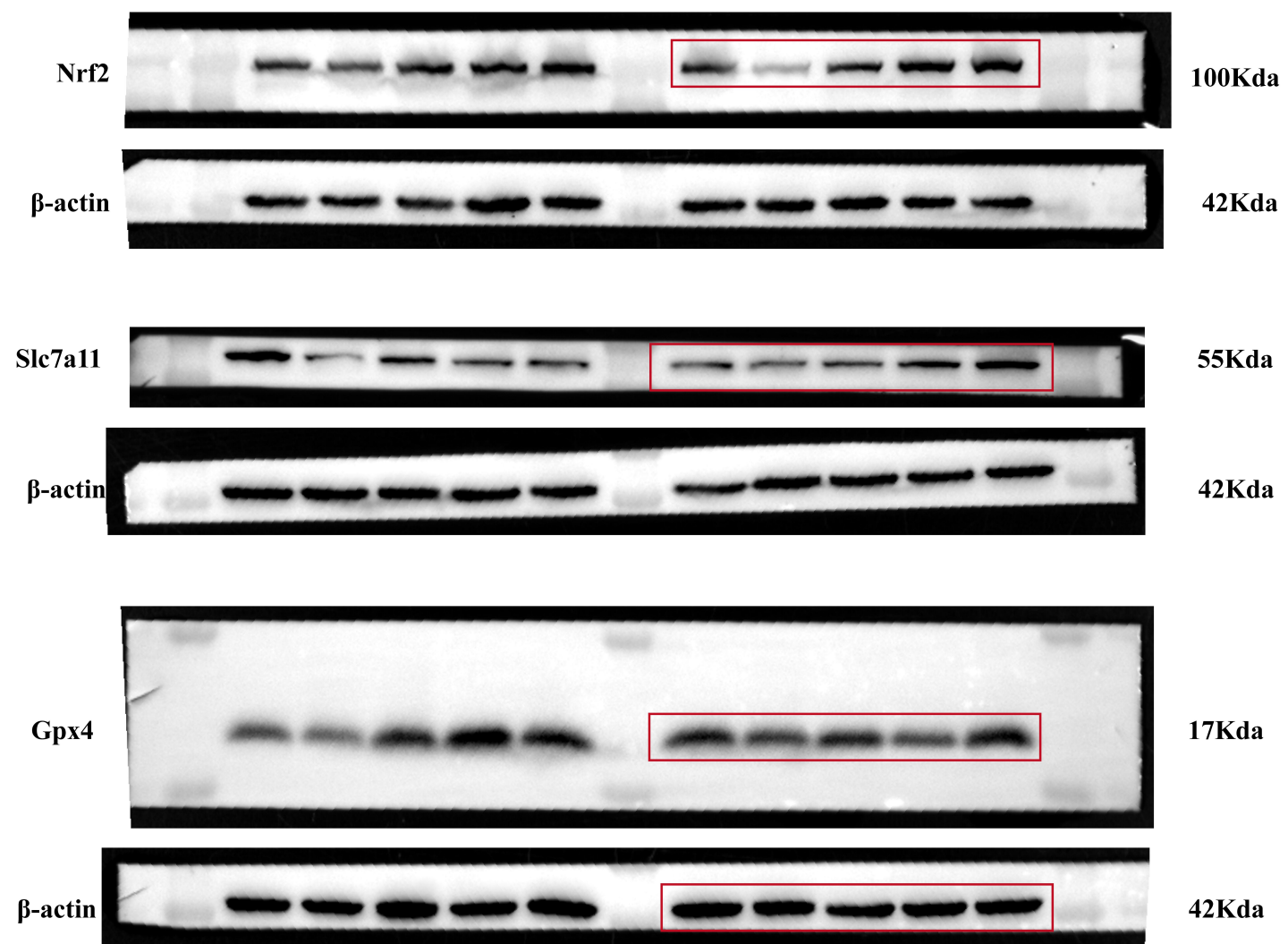

Supplement: Multimedia component 1 [file mmc1.pdf]
