# Supplementary material for: Injectable ROS homeostasis protective hydrogel inhibiting microglial ferroptosis through the Nrf2/Slc7a11/Gpx4 to alleviate neuropathic pain and promote spinal cord injury repair
Source: Redox Biol. 2025 Aug 8;86:103816. doi: 10.1016/j.redox.2025.103816 (PMC12359189; doi:10.1016/j.redox.2025.103816)
Supplement: Multimedia component 2 [file mmc2.zip › Multimedia component 2/Multimedia component 2 (revise).doc]

**Supplementary Information**

**Injectable ROS Homeostasis Protective Hydrogel Inhibiting Microglial Ferroptosis through the Nrf2/Slc7a11/Gpx4 to Alleviate Neuropathic Pain and Promote Spinal Cord Injury Repair**

Lu Li ^a, b, †^, Yu Cao^a, †^, Xiangsheng Zhang^a, †^, Jiayi Guo ^b, c^, Ziqiang Lin^a^, Pengyu Zhou^a^, Chuyin Chen^g^, Jiahao Chen^g^, Yike Liu^a^, Danzhi Luo^a^, Jiurong Chen^a^, Yingdong Deng^a^; Peng Sun^e, f *^; Zhiwen Zeng ^b, d *^; Jun Zhou^a*^

^a^ Department of Anesthesiology, The Third Affiliated Hospital, Southern Medical University, Guangzhou 510000, China,

^b^ Institute of Biological and Medical Engineering, Guangdong Academy of Sciences, Guangzhou, 510316, China,

^c^ Department of Ultrasound, Foshan Fosun Chancheng Hospital, Foshan, 528031, China

^d^ Guangdong Key Lab of Medical Electronic Instruments and Polymer Material Products, Guangzhou 510500, China

^e^ Department of Anesthesiology, Sun Yat-sen University Cancer Center, Guangzhou 510060, China.

^f^ State Key Laboratory of Oncology in South China, collaborative innovation center for cancer medicine, Guangzhou 510060, China.

^g^ ShunDe Hospital GuangZhou University of Chinese Medicine, Guangzhou 510000, China.

^†^ Co-first authors

**^*^** Corresponding Authors

Correspondence to: Jun Zhou ([zhoujun7843@smu.edu.cn](mailto:zhoujun7843@smu.edu.cn)); Zhiwen Zeng ([zengzwen@sina.cn](mailto:zengzwen@sina.cn)); Peng Sun ([sunpeng@sysucc.org.cn](mailto:sunpeng@sysucc.org.cn))

**Figure S1.** The standard curve was found by the known concentration thiol group solutions measured at 412 nm according to Ellman’s method.

**Figure S2.** Swelling ration curves of MS hydrogel and MSQ hydrogel at 10 h, (n=3).

**Figure S3.** Statistical analysis of pore size distribution in MS hydrogel (average: 1.46 μm) and MSQ hydrogel (average: 1.49 μm).

**Figure S4.** Rheological behaviors of MSQ hydrogel with different UV crosslinking time. It showed the storage modulus (G′) increased with the UV crosslinking time increasing, and then reach a steady value when the crosslinking time was 10 s.

**Figure S5.** Flow curves demonstrate the viscosity as a function of shear rate, indicating the flow behavior and injectability of the MSQ precursor solution at room temperature.

**Figure S6.** Image and schematic representation of the oxidation-reduction reactions between EGCG and antioxidant such as thiol group (-SH) and Quercetin.

**Figure S7.** (A) Superoxide anion (·O2-) scavenging ability investigation of Gel-MA, MS hydrogel and MSQ hydrogel, compared with control group, ****P<0.0001. (n=3) (B) UV−vis absorption spectra of pyrogallol aqueous solution treated with Tris·HCI solution (pH=8.0).

**Figure S8.** (A) Bubble plot shows the related pathways obtained by GO-BP gene enrichment analysis. (B) Bubble plot shows the related pathways obtained by KEGG gene enrichment analysis. (C) Venn diagram shows the intersection genes of quercetin targets, spinal cord injury related genes, neuropathic pain related genes oxidative stress related genes and ferroptosis related genes. (D-F) The top 10 genes were obtained by analyzing the 19 intersection genes by MCC, DMNC, BottleNeck and EcCentricity in CytoHubba. (G) Venn diagram shows the intersection genes of the top 10 genes of MCC, DMNC, BottleNeck and EcCentricity.

**Figure S9.** Molecular docking results of Ifng with Quercetin.

**Figure S10**. (A) 2D docking diagram of Nrf2 residues with Quercetin. (B-C) 2D and 3D binding free energy landscape of Nrf2 with Quercetin. (D) Energy profiles of Nrf2 with Quercetin. (E) Solvent-accessible surface area (SASA) results of Nrf2 with Quercetin. (F) Root mean square fluctuation (RMSF) results for Nrf2 with Quercetin.

**Figure S11.** (A-B) Immunofluorescence staining images and statistical graphs of Slc7a11 in BV2 cells, *P<0.05, **P<0.01, ***P<0.001, ****P<0.0001, scale bar = 200 μm. (n=3) (C-D) Immunofluorescence staining images and statistical graphs of Gpx4 in BV2 cells, *P<0.05, **P<0.01, ***P<0.001, ****P<0.0001, scale bar = 200 μm. (n=3)

**Figure S12. (A-C)** Determination of MDA, GSH, and Fe^2+^ levels in BV2 cells, *P<0.05, **P<0.01, ***P<0.001, ****P<0.0001, (n=3).

**Figure S13. (A-B)** Immunofluorescence staining images and statistical graphs of MitoSOX in BV2 cells, **P<0.01, ****P<0.0001, scale bar = 20 μm, (n=3).

**Figure S14.** (A-C) qPCR analysis of inflammatory factors expressions (TNFα, IL-1β, and IL-6) in BV2 cells. (D-F) qPCR analysis of M2 factors expressions (Arg1, IL-4, IL-10) in BV2 cells, *P<0.05, **P<0.01, ***P<0.001, ****P<0.0001, (n=3).

 **Figure S15.** (A-C) qPCR analysis of inflammatory factors expressions (TNFα, IL-1β, and IL-6) in BV2 cells. (D-F) qPCR analysis of M2 factors expressions (Arg1, IL-4, IL-10) in BV2 cells, *P<0.05, **P<0.01, ***P<0.001, ****P<0.0001, (n=3).

**Figure S16.** (A-B) Immunofluorescence staining images and statistical graphs of Slc7a11 in BV2 cells, *P<0.05, **P<0.01, ***P<0.001, ****P<0.0001, scale bar = 200 μm. (n=3) (C-D) Immunofluorescence staining images and statistical graphs of Gpx4 in BV2 cells, *P<0.05, **P<0.01, ***P<0.001, ****P<0.0001, scale bar = 200 μm, (n=3).

**Figure S17.** (A) qPCR analysis of IL-6 expression in BV2 cells. (B) qPCR analysis of IL-10 expression in BV2 cells, compared with Control group, ####P<0.0001, compared with LPS group, *P<0.05, ***P<0.001, ****P<0.0001, compared with LPS+MSQ group, &P<0.05. (n=3)

**Figure S18.** Quantification of abnormal mitochondria in spinal cord microglia, compared with Sham group, ####P<0.0001, compared with SCI group, ****P<0.0001, compared with MSQ group, &&&&P<0.0001. (n=6)

**Figure S19.** Immunohistochemical staining for CD86 and CD206 in spinal cord tissues from mice in each group post-SCI (scale bar = 50 μm) and statistical charts of positive cell counts, compared with the Sham group, ####P<0.0001, compared with the SCI group, **P<0.01, ***P<0.001, ****P<0.0001, compared with the MSQ group, &P<0.05, &&P<0.01, &&&P<0.001 (n=6)

**Table S1.** Protein and nucleic acid sequences of Nrf2-WT and Nrf2-MUT

**Nrf2-WT：Amino acid sequence**

MMDLELPPPGLQSQQDMDLIDILWRQDIDLGVSREVFDFSQRQKDYELEKQKKLEKERQEQLQKEQEKAFFAQFQLDEETGEFLPIQPAQHIQTDTSGSASYSQVAHIPKQDALYFEDCMQLLAETFPFVDDHESLALDIPSHAESSVFTAPHQAQSLNSSLEAAMTDLSSIEQDMEQVWQELFSIPELQCLNTENKQLADTTAVPSPEATLTEMDSNYHFYSSISSLEKEVGNCGPHFLHGFEDSFSSILSTDDASQLTSLDSNPTLNTDFGDEFYSAFIAEPSDGGSMPSSAAISQSLSELLDGTIEGCDLSLCKAFNPKHAEGTMEFNDSDSGISLNTSPSRASPEHSVESSIYGDPPPGFSDSEMEELDSAPGSVKQNGPKAQPAHSPGDTVQPLSPAQGHSAPMRESQCENTTKKEVPVSPGHQKAPFTKDKHSSRLEAHLTRDELRAKALHIPFPVEKIINLPVDDFNEMMSKEQFNEAQLALIRDIRRRGKNKVAAQNCRKRKLENIVELEQDLGHLKDEREKLLREKGENDRNLHLLKRRLSTLYLEVFSMLRDEDGKPYSPSEYSLQQTRDGNVFLVPKSKKPDTKKN

**Nrf2-MUT: Amino acid sequence**

MMDLELPPPGLQSQQDMDLIDILWRQDIDLGVSREVFDASQRQKDYELEAQKKLEKERQEQLQKEQEKAFFAQFQLDEETGEFLPIQPAQHIQTDTSGSASYSQVAHIPKQDALYFEDCMQLLAETFPFVDDHESLALDIPSHAESSVFTAPHQAQSLNSSLEAAMTDLSSIEQDMEQVWQELFSIPELQCLNTENKQLADTTAVPSPEATLTEMDSNYHFYSSISSLEKEVGNCGPHFLHGFEDSFSSILSTDDASQLTSLDSNPTLNTDFGDEFYSAFIAEPSDGGSMPSSAAISQSLSELLDGTIEGCDLSLCKAFNPKHAEGTMEFNDSDSGISLNTSPSRASPEHSVESSIYGDPPPGFSDSEMEELDSAPGSVKQNGPKAQPAHSPGDTVQPLSPAQGHSAPMRESQCENTTKKEVPVSPGHQKAPFTKDKHSSRLEAHLTRDELRAKALHIPFPVEKIINLPVDDFNEMMSKEQFNEAQLALIRDIRRRGKNKVAAQNCRKRKLENIVELAQDLGHLKDEREKLLREKGENDRNLHLLKRRLSTLYLEVFSMLRDEDGKPYSPSEYSLQQTRDGNVFLVPKSKKPDTKKN

**Table S2.** Primers used for Real-Time Quantitative PCR.

| **Gene name** | **Forward (5'⁃3')** | **Forward (5'⁃3')** |
| --- | --- | --- |
| GAPDH | TGGGTGTGAACCATGAGAAGT | AGACAATGAAATAGACGGTGGTG |
| TNFα | ACCCTGGTATGAGCCCATATAC | ACACCCATTCCCTTCACAGAGCTGAG |
| IL-1β | AAGCCTCGTGCTGTCGGACC | TGAGGCCCAAGGCCACAGGT |
| IL-6  Arg1  IL-10  IL-4 | TTGCCTTCTTGGGACTGAT  CTCCAAGCCAAAGTCCTTAGAG  CAGTACAGCCGGGAAGACAA  GGTCTCAACCCCCAGCTAGT | TTGCCATTGCACAACTCTT  AGGAGCTGTCATTAGGGACATC  CCTGGGGCATCACTTCTACC  GCCGATGATCTCTCTCAAGTGAT |

**Table S3. Target gene of quercetin from SWISS, PharmMapper, STITCH, SuperPred and ChEMBL.**

| PTGS1 | MMP1 | COL3A1 | LDHA | CECR2 | CA5A | POLB | SNX9 |
| --- | --- | --- | --- | --- | --- | --- | --- |
| AR | HIF1A | CXCL11 | CFD | NOX4 | BACE1 | NR3C2 | EPN1 |
| PTGS2 | STAT1 | CXCL2 | CA2 | GPR84 | AXL | HCK | MCL1 |
| HSP90AA1 | RUNX1T1 | DCAF5 | KRAS | SCN3A | ABCG2 | UBB | SLC2A2 |
| NCOA2 | ERBB2 | NR1I3 | ALPL | RCOR1 | NUAK1 | UBC | CYP2C8 |
| DPP4 | PPARG | CHEK2 | PGR | SLC6A5 | AKR1C2 | PTPRF | ATP5B |
| KCNH2 | ACACA | INSR | EPHX1 | AVPR2 | AKR1C1 | FABP2 | STK17B |
| SCN5A | HMOX1 | CLDN4 | LDHB | AKR1B1 | AKR1C3 | BCKDHA | HIBCH |
| ADRB2 | CYP3A4 | PPARA | TMIGD3 | MAOA | AKR1C4 | ARSB | YES1 |
| MMP3 | CYP1A2 | PPARD | THRB | IGF1R | CA13 | GRK2 | RECQL |
| F7 | CAV1 | HSF1 | ESRRA | FLT3 | AKR1A1 | CHAT | NPSR1 |
| RXRA | MYC | CRP | IMPDH2 | CYP19A1 | GPR35 | EPHB2 | HSD17B10 |
| ACHE | F3 | CXCL10 | ITGA4 | F2 | MAPT | HNF1B | IP6K2 |
| GABRA1 | GJA1 | CHUK | IDO1 | PIM1 | KDM4E | MKI67 | THRA |
| MAOB | CYP1A1 | SPP1 | NQO1 | ALOX5 | MYLK | CDKN1B | GLRA1 |
| RELA | ICAM1 | RUNX2 | NQO2 | AURKB | SYK | IQGAP1 | GPBAR1 |
| EGFR | IL1B | RASSF1 | PTPN2 | DRD4 | PIK3CG | POLR2H | MIF |
| AKT1 | CCL2 | E2F1 | DRD5 | ADORA1 | APEX1 | MAP2K6 | CDK5 |
| VEGFA | SELE | E2F2 | HRH2 | CA7 | PTPRS | BLVRA | LGALS3 |
| CCND1 | VCAM1 | ACP3 | CXCR1 | GLO1 | ESR2 | SEC24C | GUSB |
| BCL2 | PTGER3 | CTSD | CXCR2 | PIK3R1 | MPG | SEC13 | CFTR |
| BCL2L1 | CXCL8 | IGFBP3 | EDNRA | ADORA2A | SLC22A12 | RPS27A | GRIA2 |
| CDKN1A | PRKCB | IGF2 | MAPK3 | DAPK1 | CDK5R1 | UBA52 | HDAC8 |
| EIF6 | BIRC5 | CD40LG | PDE4A | PYGL | CCNB3 | SKP1 | ADRB1 |
| BAX | DUOX2 | IRF1 | PSMB9 | CA1 | ARG1 | PAFAH1B2 | MELK |
| CASP9 | NOS2 | ERBB3 | ADORA2B | GSK3B | CDK6 | ADAM17 | HDAC2 |
| PLAU | HSPB1 | PON1 | CASP1 | SRC | CDK2 | TAP1 | CHKA |
| MMP2 | TGFB1 | DIO1 | CHRNB4 | PTK2 | TYR | CAPS | MTOR |
| MMP9 | MGAM | PCOLCE | NTSR1 | HSD17B2 | HSD17B1 | GRB14 | PRKCZ |
| MAPK1 | IL2 | NPEPPS | AKT2 | KDR | APP | ERBB4 | CHRNA4 |
| IL10 | NR1I2 | HK2 | ABCC1 | MMP13 | TTR | TLR1 | TBXA2R |
| EGF | CYP1B1 | NKX3-1 | APLNR | CA3 | MMP12 | TAB1 | PTGER1 |
| RB1 | CCNB1 | RASA1 | KDM5C | ALOX15 | CD38 | SETMAR | FOSL2 |
| TNF | PLAT | GSTM1 | P2RY2 | PLK1 | AKR1B10 | SLC30A9 | MC5R |
| IL6 | THBD | GSTM2 | SLC1A2 | CA6 | TNKS2 | DNAJB8 | CPT1B |
| CDKN2A | SERPINE1 | IKBKB | CTSK | CDK1 | TNKS | GGH | SCD |
| AHSA1 | COL1A1 | CYP26A1 | HTR3A | PKN1 | TERT | CUL5 | TFPI |
| CASP3 | IFNG | CA12 | MAPKAPK2 | CA14 | APBB1 | RCHY1 | MME |
| TP53 | IL1A | KDM1A | METAP2 | CA9 | CASK | MAGI1 | GLS |
| ELK1 | MPO | PRMT3 | PRKDC | CSNK2A1 | ZEB2 | SPEN | CXCR4 |
| CAMK2B | ALK | ABCB1 | NEK6 | PLA2G1B | VPS4B | SH3BGRL | FLNB |
| RNF8 | NFAT5 | IGKC | IGHG1 | ALB | MECR | FUT8 | CELF4 |
| GAN | TDRD3 | RAB9B | ARHGEF12 | VAV3 | NEK2 | SULT1C4 | PRKD2 |
| NFKBIA | POR | ODC1 | XDH | CASP8 | TOP1 | RAF1 | SOD1 |
| PRKCA | TOP2A | NCF1 | HAS2 | GSTP1 | NFE2L2 | PARP1 | AHR |
| PSMD3 | SLC2A4 | ICMT | BRD4 | SOAT2 | PDE5A | PDE9A | S1PR2 |
| PDE8B | TDP2 | BRD1 | PTPN11 | PDE4B | PIN1 | PTK6 | TPSAB1 |
| ATAD2 | KISS1R | P2RX4 | MKNK1 | ALOX12 | MET | CA4 |  |

**Table S4.** TOP 10 genes in different algorithms of CytoHubba.

| **MCC** | **DMNC** | **BottleNeck** | **EcCentricity** |
| --- | --- | --- | --- |
| Il6 | Ifng | Src | Il6 |
| Src | Ppara | Gsk3b | Src |
| Mtor | Nfe2l2 | Nos2 | Nos2 |
| Pparg | Nos2 | Nfe2l2 | Nfe2l2 |
| Il1b | Pparg | Ifng | Ifng |
| Ifng | Mtor | Ar | Ar |
| Ppara | Rela | Cav1 | Cav1 |
| Rela | Kras | Prkca | Prkca |
| Nfe2l2 | Tert | Tert | Tert |
| Kras | Il1b | Gja1 | Gja1 |
